# DockM8: an all-in-one open-source platform for consensus virtual screening in drug design

**DOI:** 10.1186/s13321-026-01287-2

**Published:** 2026-09-18

**Authors:** Antoine Lacour, Hamza Agha, Anna K. H. Hirsch, Andrea Volkamer

**Affiliations:** 1 Helmholtz Institute for Pharmaceutical Research Saarland (HIPS), Helmholtz Centre for Infection Research (HZI), Campus E8.1, 66123 Saarbrücken, Saarland Germany; 2 Saarland University, Department of Pharmacy, Campus E8.1, 66123 Saarbrücken, Saarland Germany; 3 Data Driven Drug Design, Faculty of Mathematics and Computer Sciences, Saarland University, Campus E2.1, 66123 Saarbrücken, Saarland Germany; 4 PharmaScienceHub, Saarland University, Campus E2.1, Saarland 66123 Saarbrücken, Germany

**Keywords:** Virtual screening, Docking, Consensus, Open-source

## Abstract

Virtual screening remains a critical step in structure-based drug design, yet variability in docking algorithms and scoring functions often limits its reliability. To address this challenge, we introduce DockM8, an open-source platform for consensus virtual screening that integrates pocket detection, ligand preparation, docking, rescoring, and consensus ranking within a single modular workflow. DockM8 supports five docking engines, 17 pose-selection methods, 17 scoring functions (SFs), and five consensus methods, all accessible through both a graphical user interface (GUI) and a Python application programming interface (API). Systematic evaluation on the DEKOIS 2.0, DUD-E, and Lit-PCBA datasets yielded median relative enrichments of 100%, 82.59%, and 8.85%, respectively, frequently matching or outperforming state-of-the-art approaches. Our findings further reveal that no pose-selection or consensus strategy universally excels, emphasizing the need for tailored, target-specific workflows. DockM8 is freely available under the GNU General Public License at https://github.com/DrugBud-Suite/DockM8.

**Scientific contribution**

DockM8 is, to our knowledge, the most comprehensive open-source platform to integrate the complete consensus structure-based virtual screening (SBVS) pipeline within a single, graphical-interface-driven tool, yielding over 55 million configurable protocols, a breadth unmatched by previously published consensus docking software. Through systematic multi-benchmark evaluation, we demonstrate that no single pose-selection or consensus strategy generalizes across targets, motivating a target-specific workflow-selection paradigm in place of prevailing one-size-fits-all heuristics. By delivering this tunability through a graphical interface rather than scripts, DockM8 brings rigorous, reproducible consensus virtual screening within reach of non-specialist medicinal chemists.

**Graphic Abstract:**

**Figure:**
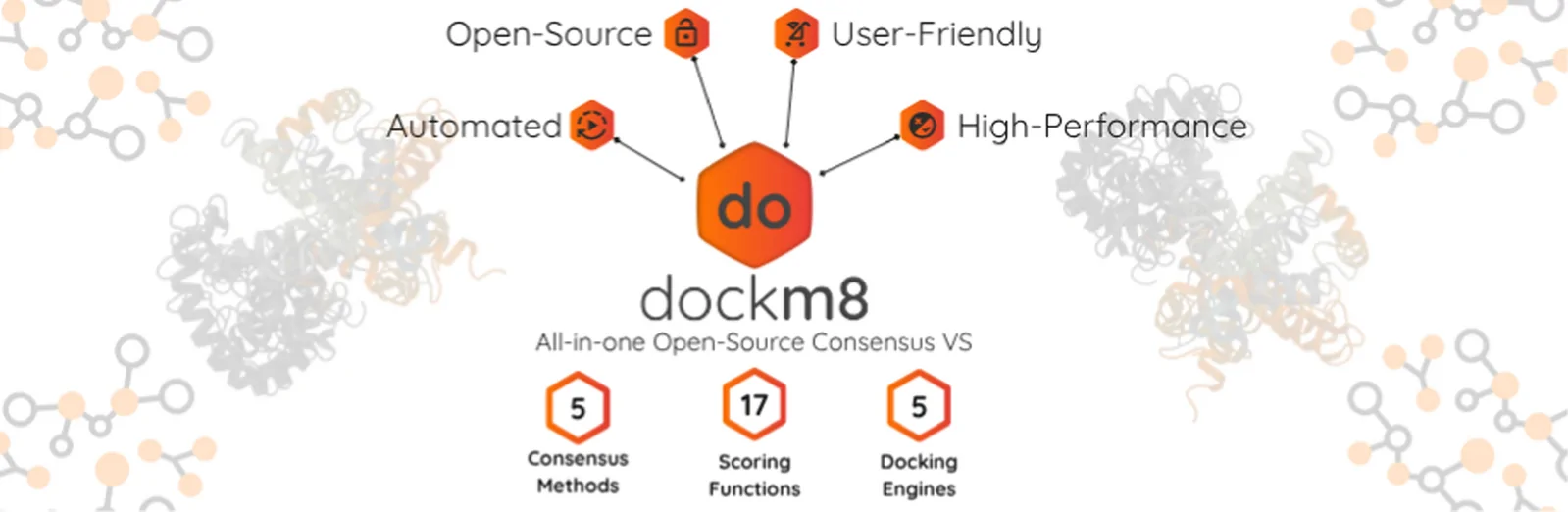

**Supplementary Information:**

The online version contains supplementary material available at https://doi.org/10.1186/s13321-026-01287-2.

## Introduction

In the pursuit of accelerating drug discovery, computational methods have become indispensable, with SBVS emerging as a cornerstone strategy. Docking-based virtual screening (VS) uses 3D structural information and computational algorithms to sift through large molecular libraries, identifying compounds with potential biological affinity for a specified target. Consequently, this screening reduces the number of compounds subjected to “wet-lab” assays, substantially curbing associated costs while hoping to discover novel active compounds [1, 2].

Molecular docking simulates the interaction between a ligand and its target, attempting to predict the binding pose of the ligand of interest and computing a docking score used to rank potential ligands based on predicted binding affinities [3]. Determining the accurate binding pose of a small molecule is essential not only for assessing its binding affinity but also for leveraging the pose in lead optimization. Furthermore, accurate scoring of compounds is crucial for comparing ligands in a screening scenario and determining whether a particular ligand should be considered for experimental validation.

Conventional molecular docking software, including AutoDock [4], AutoDock Vina [5, 6], and Glide [7], predominantly employ heuristic search algorithms to systematically examine a range of potential ligand conformations. Although the sampling of binding poses performed by these algorithms has seen significant advances in recent years, particularly with recent developments, challenges in scoring and ranking the resulting poses still remain due to known shortcomings such as not considering protein flexibility, water-mediated interactions, or changes in entropy upon binding.

Over the past decades, a multitude of SFs have been developed, ranging from empirical, physics-based, and knowledge-based SFs to the more recently developed machine-learning (ML) based SFs [8, 9]. Despite significant recent advances in this field, these SFs still lack universal applicability as they necessarily rely on a simplification of the complexity of protein–ligand binding. Indeed, the performance of any given SF on a given target does not necessarily correlate to performance on other targets [10]. Moreover, single SFs often struggle to handle both accurate binding affinity prediction and ligand ranking [11]. Several solutions have been proposed to deal with the issues surrounding the performance of molecular docking, including consensus docking (using multiple methods) and ensemble docking (incorporating several protein conformations).

Ensemble docking uses multiple receptor conformations to tackle the inherent challenge of protein flexibility. Indeed, early SBVS campaigns are mostly performed using flexible ligands and rigid receptor docking. Although this accelerates the calculation, it fails to represent the dynamics of the protein–ligand system under study. Ensemble docking suggests that using multiple protein states instead of a single static structure offers a more holistic view of ligand binding. This approach improves the predictive accuracy of molecular docking by considering scores and rankings across these various protein states [12].

Consensus docking has also emerged as a modality seeking to enhance the precision of molecular docking. By combining diverse docking algorithms and SFs, consensus approaches circumvent individual limitations through aggregation. This strategy was shown to yield more reliable binding pose predictions, better absolute and relative scoring of ligands, and superior generalizability [13–16].

In recent years, a variety of consensus docking tools have been developed (Table 1). DockBox uses a variety of docking algorithms (AutoDock4 [4], Vina [5], and DOCK [17]) along with score-based consensus docking to enhance pose prediction and ligand ranking [18]. dockECR uses LeDock [19], rDock [20], Smina [21] and Vina along with exponential consensus ranking (ECR) to improve binding pose prediction [22]. Approaches such as MetaDOCK [23], CompScore [24] or ESSENCE-Dock [25] attempt to find novel methods of carrying out the consensus by using docking-pose based clustering, incorporating additional SF components in the consensus, or using pose root-mean-square deviation (RMSD) and the number of rotatable bonds to further refine the consensus scoring. Also recently, DockingPie [26] offers a GUI in PyMOL [27] to aid in consensus screening.

**Table 1:** Comparison of docking tools

Tool (Year)	Open source	Docking	GUI or server	Methods		No external licenses
				C	S	
VoteDock [28] (2011)	––	x	––	1	> 5	––
CompScore [24] (2019)	x	––	x	1	0	x
DockBox [18] (2019)	x	x	–	2	5	––
dockECR [22] (2021)	x	x	––	1	4	––
DockStream [29] (2021)	x	x	––	0	> 5	––
pyscreener [30] (2021)	x	x	––	0	> 5	x
DockingPie [26] (2022)	x	x	x	1	4	––
MetaDOCK [23] (2023)	––	x	x	1	3	x
E-DOCK [25] (2023)	x	––	––	1	3	––
ChemFlow [31] (2023)	x	x	––	0	3	––
easydock [32] (2023)	x	x	––	0	>2	––
Dockey [33] (2023)	x	x	x	0	3	––
DockM8 (ours) (2026)	x	x	x	5	17	x

‘x’ means respective column applies to this tool, ‘–'‘–’ not applicable. The more x’s the better

E-DOCK: ESSENCE-DOCK, GUI: graphical user interface, C: consensus methods, S: scoring functions

Despite this proliferation of consensus docking tools (Table 1), many do not offer graphical interfaces or web servers, which can limit accessibility for users less familiar with command-line workflows. Additionally, some tools provide a limited selection of consensus methods or SFs, restricting their adaptability to different docking scenarios. Several also require manual compilation or obtaining software from various commercial or academic sources, which may hinder widespread adoption in academic drug discovery workflows.

**Fig. 1:**
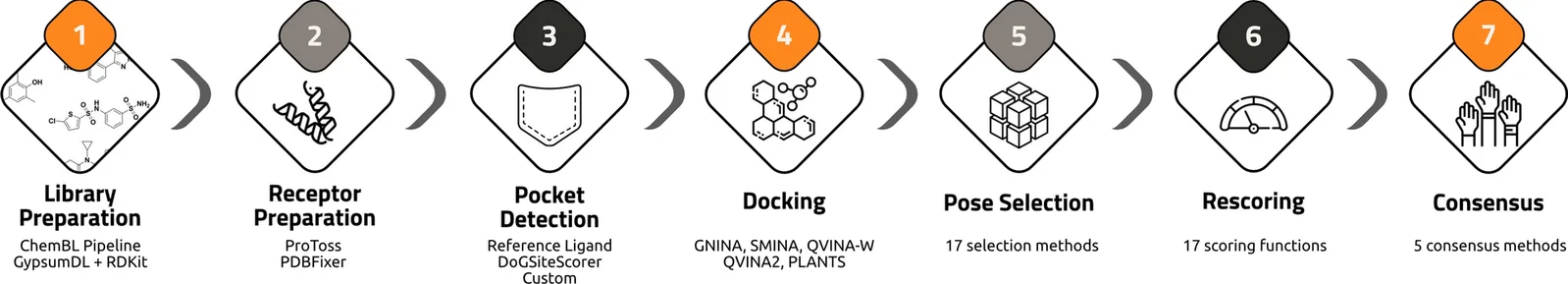
General workflow for consensus docking and scoring within DockM8. The seven-step workflow is shown, along with the various tools available to the user

To address these shortcomings, we developed DockM8, a comprehensive open-source workflow for consensus VS that encompasses the entire SBVS pipeline. DockM8 integrates various programs and APIs for protein and ligand preparation, binding site determination, docking, pose-selection, rescoring, and consensus scoring (Fig. 1). It supports both single and ensemble docking modes with up to five docking tools, 17 pose-selection methods, and 17 SFs (section Methods). With the addition of five consensus methodologies and DockM8’s modular architecture, the platform can generate over 55 million distinct workflow configurations. This extensive configurability enables both target-specific optimization and cross-target generalizability assessments. Designed for ease of use by both computational and medicinal chemists, DockM8 can be run through a GUI, command line, or Jupyter Notebooks.

In this work, we introduce DockM8 and extensively evaluate its performance on subsets of the Directory of Useful Decoys-Enhanced (DUD-E) [34], Demanding Evaluation Kits for Objective In Silico Screening (DEKOIS) [35], and Lit-PCBA [36] datasets, demonstrating its consistent effectiveness across diverse targets and its capacity to tailor virtual screening protocols beyond the capabilities of individual scoring functions.

## Results and discussion

### Assessment of screening power on DEKOIS 2.0, DUD-E and Lit-PCBA

**Fig. 2:**
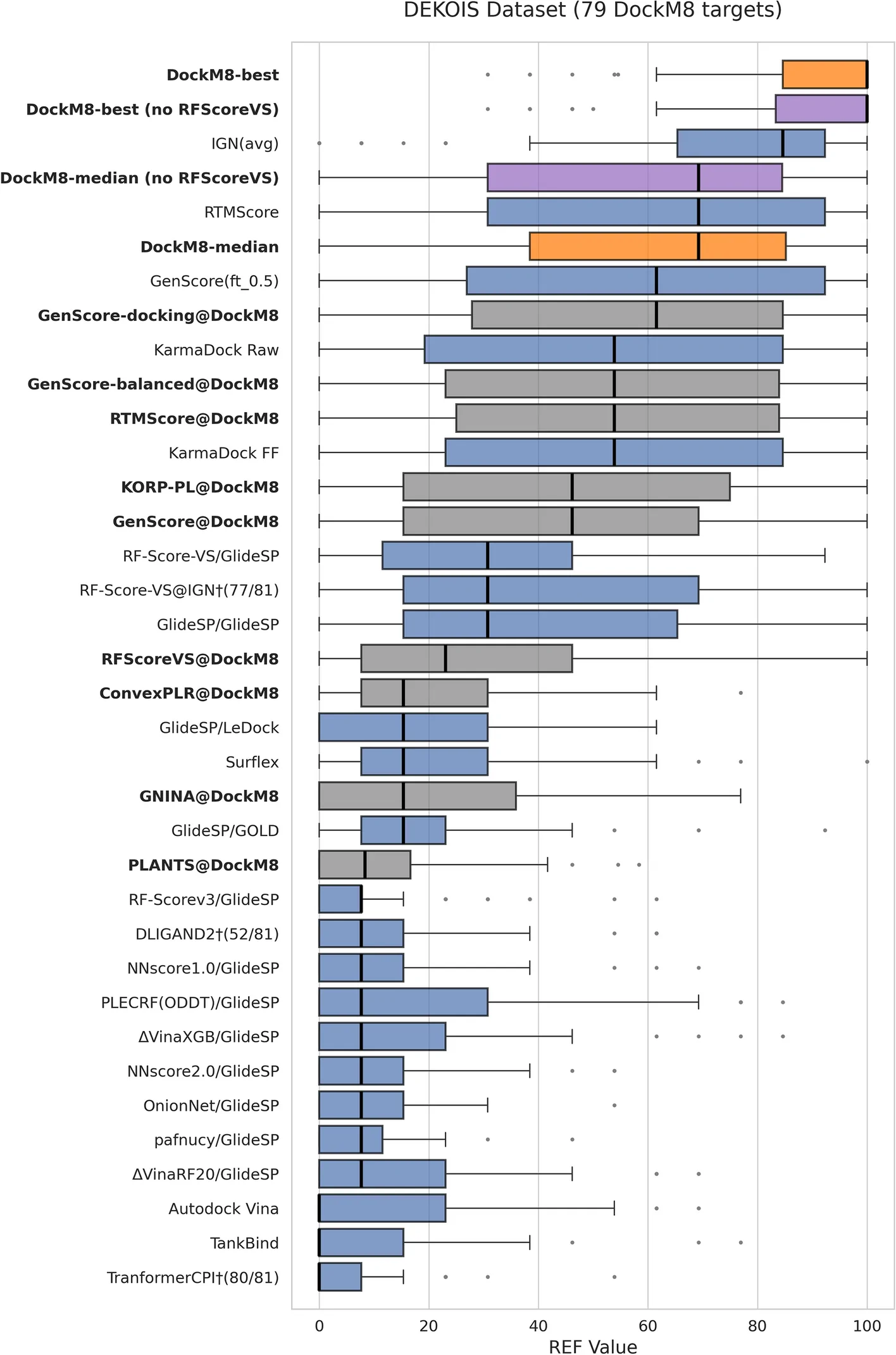
Evaluation of $\hbox {REF}_{1\%}$ of DockM8 compared to published protocols on the DEKOIS dataset. Methods are sorted by their median value. Whiskers denote the 10th and 90th percentiles. Methods containing the @ symbol refer to the provenance of the data (@IGN) while methods containing the/symbol refer to software used to obtain the docking poses which were used for scoring (/GlideSP) and performance assessment. Methods tested on only a subset of targets are highlighted with † and the number of targets tested is shown in brackets. DockM8-best and DockM8-median (including RF-Score-VS) are shown in orange, their RF-Score-VS-excluded variants (-noRFScoreVS) in purple, and the individual scoring functions evaluated within the DockM8 framework (the @DockM8 methods) in grey; the remaining entries are published literature protocols. The set of literature comparators differs between datasets because not all methods were reported on all three benchmarks

**Fig. 3:**
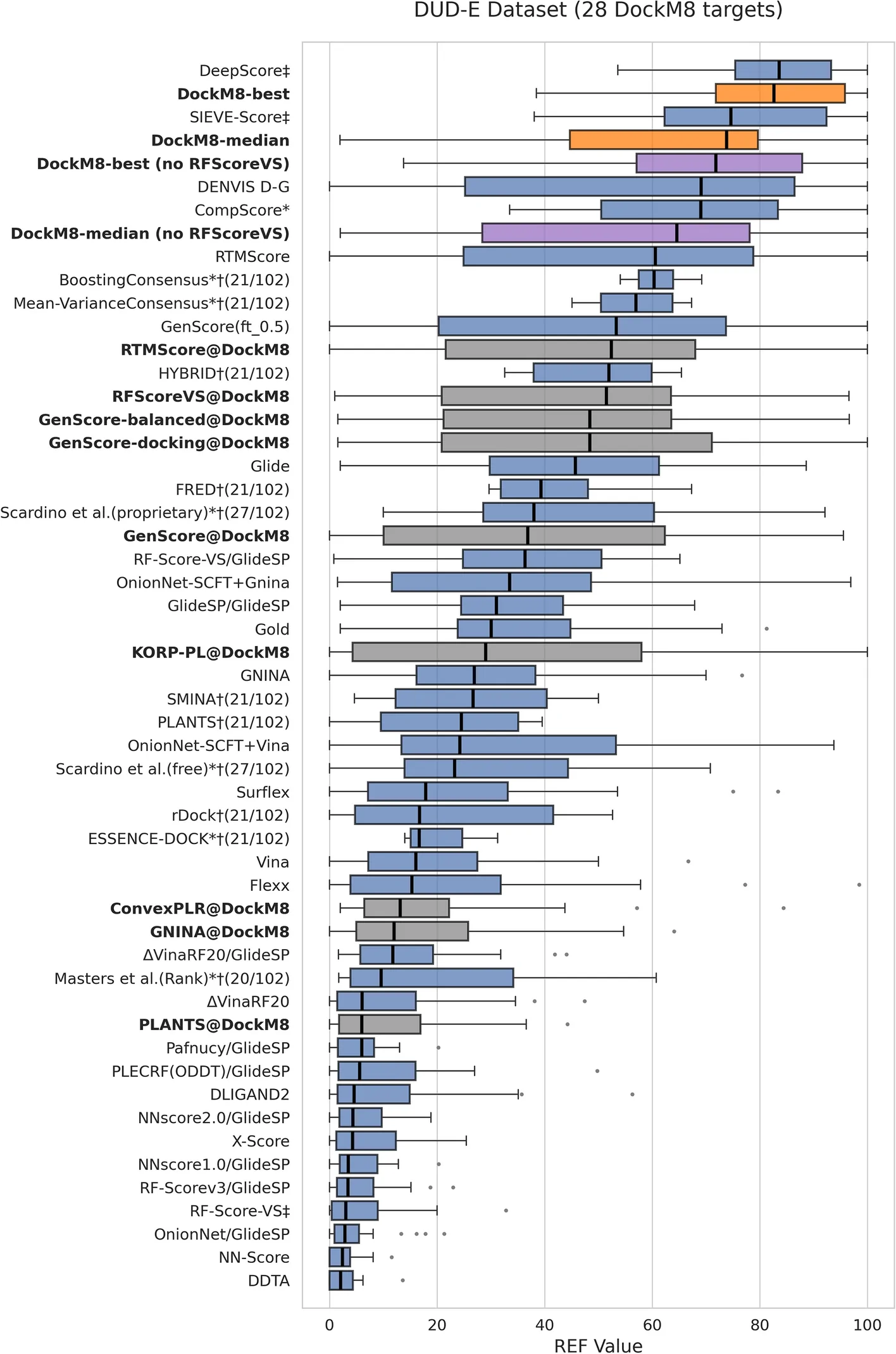
Evaluation of $\hbox {REF}_{1\%}$ of DockM8 compared to published protocols on the DUD-E dataset. Methods are sorted by their median value. Whiskers denote the 10th and 90th percentiles. Methods containing the @ symbol refer to the provenance of the data (@DockM8) while methods containing the/symbol refer to software used to obtain the docking poses which were used for scoring (/GlideSP) and performance assessment. Methods that use a consensus approach are highlighted with *. Methods tested on only a subset of targets are highlighted with † and the number of targets tested is shown in brackets. Methods that used the same dataset for training are highlighted with ‡. DockM8-best and DockM8-median (including RF-Score-VS) are shown in orange, their RF-Score-VS-excluded variants (-noRFScoreVS) in purple, and the individual scoring functions evaluated within the DockM8 framework (the @DockM8 methods) in grey; the remaining entries are published literature protocols. The set of literature comparators differs between datasets because not all methods were reported on all three benchmarks

**Fig. 4:**
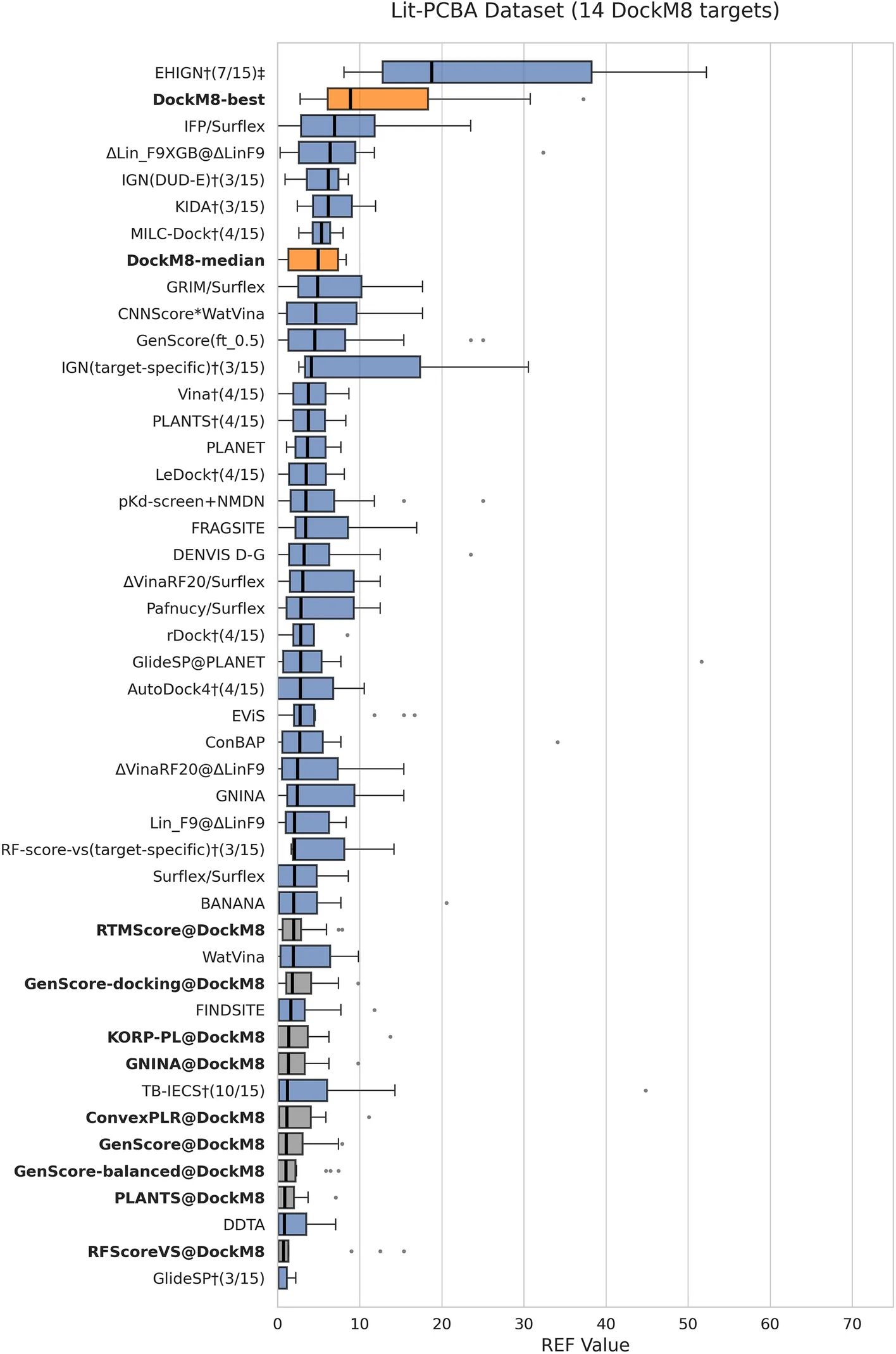
Evaluation of $\hbox {REF}_{1\%}$ of DockM8 compared to published protocols on the Lit-PCBA dataset. Methods are sorted by their median value. Whiskers denote the 10th and 90th percentiles. Methods containing the @ symbol refer to the provenance of the data (@PLANET) while methods containing the/symbol refer to software used to obtain the docking poses which were used for scoring (/GlideSP) and performance assessment. Methods that use a consensus approach are highlighted with *. Methods tested on only a subset of targets are highlighted with † and the number of targets tested is shown in brackets. Methods that used the same dataset for training are highlighted with ‡. DockM8-best and DockM8-median are shown in orange, and the individual scoring functions evaluated within the DockM8 framework (the @DockM8 methods) in grey; the remaining entries are published literature protocols. The set of literature comparators differs between datasets because not all methods were reported on all three benchmarks

To evaluate the performance of DockM8 against established methods in SBVS, we conducted a comprehensive comparison using three benchmark datasets: DEKOIS 2.0 (79 targets), DUD-E (28 targets), and Lit-PCBA (14 targets). Performance was assessed using Relative Enrichment Factor $\hbox {(REF)}_{1\%}$, which normalizes enrichment to the theoretical maximum achievable for each target, thereby providing a standardized measure across different datasets, targets, and methods. Figures 2, 3, and 4 present the comparative performance across all three datasets. Supplementary comparisons using additional metrics (AUC-ROC, BEDROC, EF, PM) and extended model/target sets are presented in Figures S1–S14 of Additional file 1. For the rest of this discussion, we define a "DockM8 workflow" as a unique combination of a pose-selection method, a consensus method, and one or more SFs. Two operational modes of DockM8 were evaluated: (1) DockM8-best, representing the optimal combination of scoring, docking, pose-selection, and consensus methods for each individual target; and (2) DockM8-median, denoting the single workflow configuration that achieved the highest median $\hbox {REF}_{1\%}$ across all targets within a dataset (i.e., one fixed combination of docking engine, pose-selection method, consensus method, and scoring functions per dataset; the resulting configurations are listed in Appendix F). Additionally, we included the performance of individual SFs within the DockM8 framework (denoted as "@DockM8") to provide a more equitable comparative basis. For example, while RTMScore represents performance data reported in the original literature, RTMScore@DockM8 indicates the performance we obtained using that same SF but with our specific pose generation, protein preparation, and library preparation protocols. This distinction enables a fairer assessment of the contribution of our consensus approach relative to single SFs, as both use identical input data and preparation methods.

DEKOIS 2.0 Dataset: On the DEKOIS dataset, DockM8-best outperformed all other methods, demonstrating the potential of customized workflows for individual targets (median $\hbox {REF}_{1\%}$ of 100%). Additionally, the method never showed a $\hbox {REF}_{1\%}$ of less than 30% with it obtaining an $\hbox {REF}_{1\%}$ of 30.77% for the 17BETAHSD1 target. This is in contrast to even the state-of-the-art methods such as IGN [37] and RTMScore/GlideSP, where the “/” notation denotes the software used to generate the docking poses (here GlideSP [7]) that were then scored with a specific scoring function (here RTMScore [11]), showing poor performance on certain targets (some targets have $\hbox {REF}_{1\%}$ of 0%). Notably, DockM8-median exhibited superior performance compared to most literature methods (median $\hbox {REF}_{1\%}$ of 69.23%), positioning itself among state-of-the-art SFs such as RTMScore [11] and GenScore [38]. This indicates that a single, well-configured DockM8 workflow can achieve robust performance across diverse targets. As a precaution against possible training-set overlap, we additionally report DEKOIS performance with RF-Score-VS excluded; this leaves the results essentially unchanged (DockM8-best and DockM8-median medians of 100% and 69.23%, respectively; Fig. 2).

DUD-E Dataset: For the DUD-E dataset, we conducted analyses both with and without RF-Score-VS, as this SF was partially trained on DUD-E data, potentially introducing bias. DockM8-best achieved a median $\hbox {REF}_{1\%}$ of 82.59% and showed decent enrichment across all targets, with the lowest $\hbox {REF}_{1\%}$ obtained being 38.46% for the HDAC2 target. DockM8-best also outperformed DeepScore [39] and SIEVE-Score [40], state-of-the-art methods that were partially trained using DUD-E data. Even with the exclusion of RF-Score-VS, DockM8-best-noRFScoreVS (median $\hbox {REF}_{1\%}$ of 71.84%) maintained superior performance compared to all literature methods, also demonstrating consistent performance with its lowest $\hbox {REF}_{1\%}$ being 13.78% for the DHI1 target. The performance difference between DockM8-best modes and literature methods was particularly pronounced, emphasizing the substantial benefits of workflow customization for specific targets. Moreover, DockM8-median-noRFScoreVS demonstrated higher performance than RFScoreVS@DockM8, further validating the efficacy of the consensus approach. DockM8-median achieved a median $\hbox {REF}_{1\%}$ of 73.80% across DUD-E targets, although its weakest target, HDAC2, reached only 1.94%. Excluding RF-Score-VS (DockM8-median-noRFScoreVS lowered the median to 64.58% and shifted the weakest target to DHI1 (2.04%).

Lit-PCBA Dataset: The Lit-PCBA dataset presents a particularly challenging benchmark due to the use of more realistic experimental bioassay data [36]. On this dataset, DockM8-best maintained its superior performance relative to a majority of literature methods, achieving a median $\hbox {REF}_{1\%}$ of 8.85% and a lowest $\hbox {REF}_{1\%}$ of 2.73% for the VDR target. We note that the only method outperforming DockM8-best was EHIGN [41], which was partially trained on Lit-PCBA data. DockM8-median achieved a median $\hbox {REF}_{1\%}$ of 4.93% and showed a worst performance of 0% $\hbox {REF}_{1\%}$ for the MTORC1 target. These generally lower $\hbox {REF}_{1\%}$ values compared to DEKOIS and DUD-E are indicative of the more challenging nature of this dataset. However, the performance of DockM8-best on a challenging, realistic dataset is especially important as it demonstrates the utility of tailoring a consensus method to a specific target, particularly given that for some targets, high enrichments could still be achieved (e.g., DockM8-best achieved 37.26% $\hbox {REF}_{1\%}$ for ESR1T). DockM8-median demonstrated competitive performance relative to established literature methods, consistently ranking among the top-performing approaches. This outcome underscores the robustness of the DockM8 framework across diverse target classes.

Across all datasets, a noticeable discrepancy was observed between the performance reported in the original literature for certain SFs (e.g., GenScore(ft0.5), RF-Score-VS/GlideSP) and the performance achieved when these same functions were invoked via the DockM8 framework (e.g., GenScore@DockM8, RFScoreVS@DockM8), as depicted in Figs. 2, 3, 4. These variations likely stem from differences in the underlying methodologies used for protein preparation, ligand preparation, binding site selection, pose generation, and pose-selection strategies between the original studies and our standardized DockM8 protocol. This underscores the critical impact of the entire VS workflow, beyond just the SF itself, on the final outcome. When comparing strictly within our own framework (i.e., SFs applied to the same poses generated through our protocol), Figs.  10,  11, and  12 (Appendix A) demonstrate that our consensus approaches (DockM8-best and DockM8-median) consistently outperform individual SFs across all three benchmark datasets.

The strong, target-specific performance of DockM8-best across all three benchmark datasets represents a significant advancement in SBVS methodology, underscoring the substantial performance gains achievable through tailoring consensus workflows to the target under study. We emphasize, however, that DockM8-best is by construction the configuration selected *a posteriori* as optimal for each individual target from among the many evaluated, and therefore represents an upper bound on achievable performance rather than a configuration that could be selected prospectively; we investigate data-driven, prospective selection of a workflow from a small calibration set of known actives in a separate study [42]. However, it is important to note that this analysis was conducted retrospectively on benchmark datasets. Without prior inhibition or activity data, it would not be feasible to determine the optimal DockM8-best configuration for a new target. The question of whether the identified optimal workflow for a specific target would maintain its performance upon the introduction of new data will be addressed in section Generalizability assessment and predictive capability of DockM8 workflows.

The strong performance of DockM8-median (with median $\hbox {REF}_{1\%}$ values of 69.23%, 73.80%, and 4.93% on DEKOIS, DUD-E, and Lit-PCBA, respectively) across all datasets demonstrates that a single DockM8 configuration can achieve competitive performance across diverse targets without target-specific optimization. This finding has significant implications for practical applications of SBVS, suggesting that DockM8 can serve as a strong default starting point for VS campaigns against novel targets. This is particularly evident when comparing DockM8-median to individual SFs (@DockM8 methods) operating under identical conditions (same poses, library preparation, and protein preparation) (Figs. 10, 11, 12, Appendix A). The consistent improvement of DockM8-median over individual SFs provides compelling evidence for the efficacy of consensus methods and SF combinations in enhancing VS performance.

In conclusion, our evaluation demonstrates that DockM8 achieves competitive performance in SBVS, reaching state-of-the-art results on the synthetic DEKOIS 2.0 and DUD-E benchmarks, while screening power varies substantially across datasets and targets. The framework’s flexibility in allowing customization for specific targets (DockM8-best) and its robust performance with a single configuration (DockM8-median) represent significant advancements in addressing the long-standing challenge of developing generalizable screening methods in computer-aided drug design.

### Generalizability assessment and predictive capability of DockM8 workflows

**Fig. 5:**
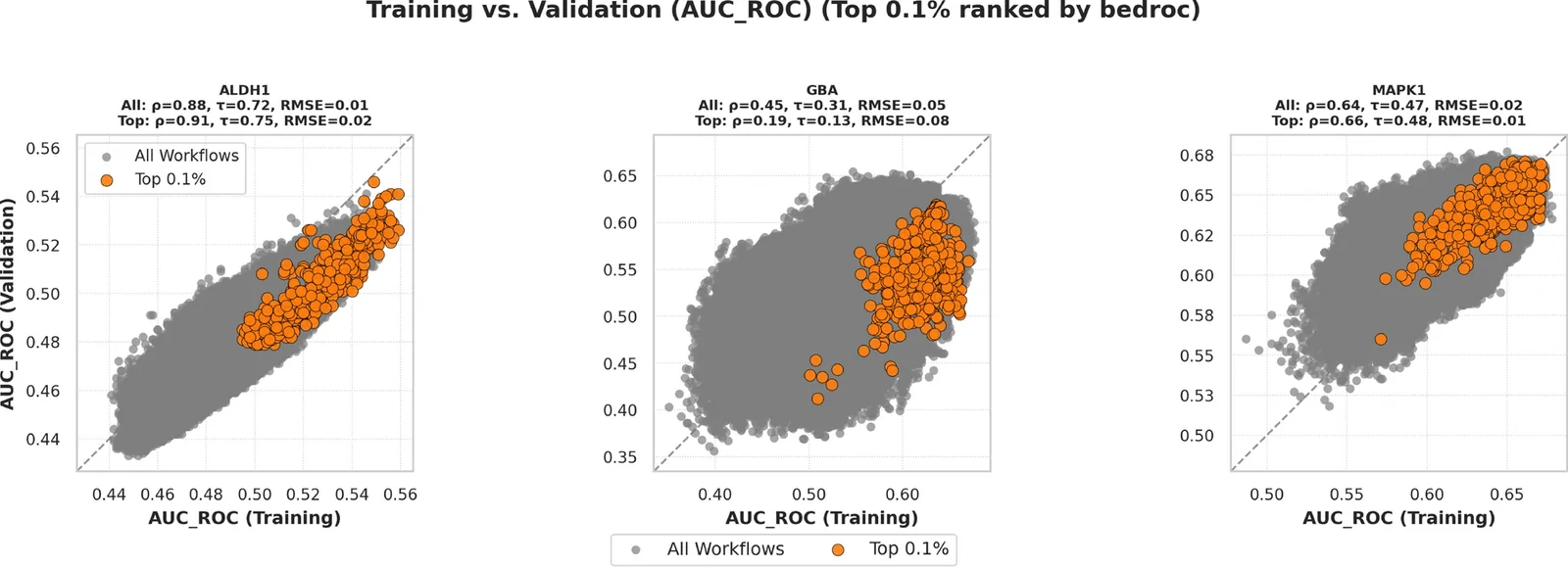
Training vs Validation Area under the Receiver Operating Characteristic Curve (AUC-ROC) performance across three representative Lit-PCBA targets using AVE splits. Each point represents a unique workflow configuration with gray circles showing all workflows and orange points highlighting the top 0.1% of workflows ranked by training-set Boltzmann-Enhanced Discrimination of ROC (BEDROC) score. For each target, correlation metrics (Spearman’s ρ, Kendall’s τ, and RMSE) are displayed separately for all workflows (All) and the top 0.1% subset (Top). Results for all 14 targets are shown in Supplementary Figure S15

Assessing the generalizability of complex virtual screening workflows beyond benchmark datasets is essential. To prospectively assess the ability of high-performing DockM8 workflows to generalize to new data, we utilized the AVE splits of the Lit-PCBA dataset (see section Asymmetric validation embedding analysis). We evaluated workflow performance by comparing their AUC-ROC values between the training and validation sets for all 14 Lit-PCBA targets (Fig. 5, Supplementary Figure S15). For each target, we evaluated the Spearman’s correlation coefficient (ρ), Kendall’s rank correlation coefficient (τ), and Root Mean Square Error (RMSE) by comparing each workflow’s AUC-ROC score on the training set against its AUC-ROC on the validation set. The correlation coefficients assess whether workflow rankings are preserved across sets, while RMSE measures the magnitude of score differences from perfect agreement. In addition to the full set of workflows, we highlight each target’s top-performing workflows, namely the top 0.1% shown in Fig. 5, defined by ranking workflows on their training-set BEDROC (a metric that rewards early recognition of actives and so isolates the configurations a screener would prioritize). We report ρ and τ separately for all workflows and for this training-selected subset; the latter tests whether selecting the best-scoring workflows on the training set carries over to the validation set. Note that the ranking used to define these subsets (training-set BEDROC) is distinct from the quantity being correlated (training versus validation AUC-ROC).

The analysis revealed a broad spectrum of generalizability across targets. Among the three representative targets in Fig. 5, agreement between the training and validation rankings was strong for ALDH1, weaker for GBA, and moderate for MAPK1. Across all workflows the Spearman coefficients were ρ=0.88, 0.45, and 0.64 (Kendall’s τ=0.72, 0.31, and 0.47), respectively; within each target’s top 0.1% subset they were ρ=0.91, 0.19, and 0.66 (τ=0.75, 0.13, and 0.48). For ALDH1 and MAPK1, these top 0.1% workflows (orange points in Fig. 5) clustered in the high-performance region on validation, whereas for GBA they did not. Indeed, for GBA the agreement was lower within the top 0.1% subset than across all workflows, because the configurations ranked best in training did not retain their advantage on validation. Across all 14 targets (Supplementary Figure S15), agreement over all workflows ranged from strong (ALDH1 ρ=0.88, MAPK1 ρ=0.64, TP53 ρ=0.50) through weak or near-zero (ESR1T ρ=0.09, KAT2A ρ=-0.02) to negative (IDH1 ρ=-0.31, PPARG ρ=-0.19, MTORC1 ρ=-0.11, ADRB2 ρ=-0.07). For the negatively correlated targets, training-set performance was not predictive of validation performance. This variability was associated with each target’s active-to-inactive ratio. Among the top-performing (top 0.1%) workflows, agreement between training and validation increased with this ratio (Spearman ρ=0.74, p=0.002; Supplementary Figure S16). Targets with a high proportion of actives, such as ALDH1, gave more robust and more transferable rankings than active-sparse targets such as ADRB2 and IDH1, for which too few positives were available for a stable ranking to emerge. Intrinsic differences in how amenable each target is to docking are also likely to contribute.

In conclusion, this AVE analysis provides nuanced evidence for the generalizability of optimized DockM8 workflows. While perfect correlation between training and validation performance cannot be expected, the results demonstrate that performance trends identified through rigorous evaluation can translate to new data for some targets, particularly those with sufficient active compounds, but for several others (including the targets with negative or near-zero correlation noted above), training-set performance was not predictive. This supports workflow customization, as facilitated by DockM8, as a rational but not universally reliable strategy for enhancing virtual screening effectiveness in practical drug discovery efforts.

### Evaluation of component performance across datasets

**Fig. 6:**
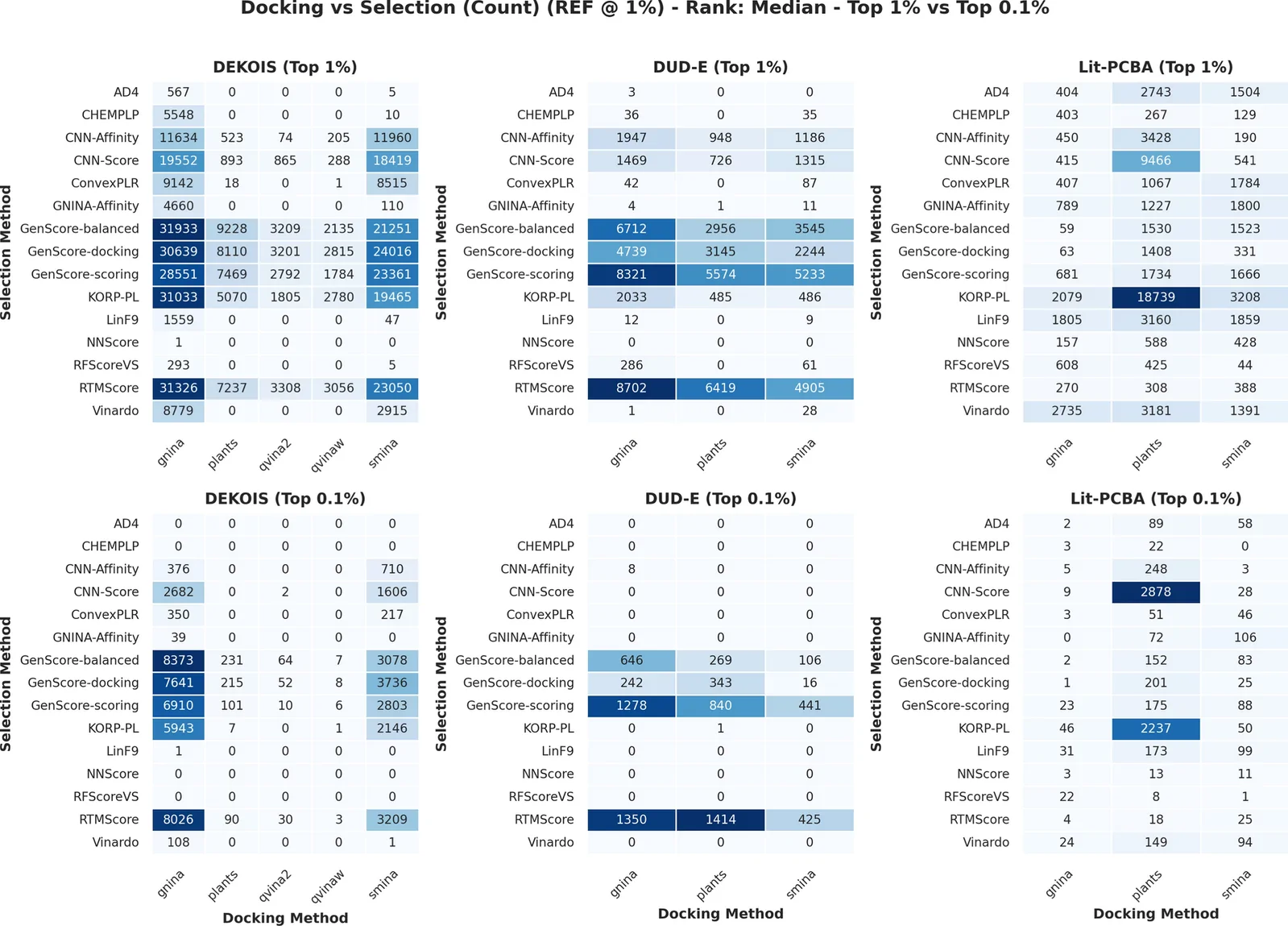
Docking and pose-selection method frequency in top-performing DockM8 workflows. Frequency (by absolute count) at top performance thresholds (1%, 0.1%) on the DEKOIS, DUD-E, and Lit-PCBA datasets

Our comprehensive analysis of the contributions of individual components (docking engines, pose-selection methods, consensus approaches, and nature and number of SFs) reveals distinct patterns of performance across different benchmark datasets, offering valuable insights for both methodological development and practical application of SBVS. We evaluated the impact of the selection of consensus methods, pose-selection methods, SF selection, and the number of SFs used during the consensus procedure on DockM8’s performance. For each DockM8 combination, we computed the median performance across all targets within the given dataset, using the $\hbox {REF}_{1\%}$ metric as a standard measure.

To assess the prevalence of specific pose-selection strategies, consensus methods, and scoring functions (SFs) among the top-performing DockM8 workflows, we quantified how frequently each appeared within the highest-ranked subset based on the median $\hbox {REF}_{1\%}$ metric across targets. Rankings were determined at multiple performance thresholds; for example, in a set of 1000 DockM8 workflows, the top 1% threshold corresponds to the 10 workflows with the highest $\hbox {REF}_{1\%}$ values. This threshold-based approach was deliberately chosen over calculating the median or mean performance of each method across the entire dataset, as the latter may obscure top-level performance and disproportionately favor methods that excel on isolated targets.

#### Docking and pose-selection

Figure 6 summarizes the frequency with which specific docking- and pose-selection methods appeared among the top-performing DockM8 workflows on the DEKOIS, DUD-E, and Lit-PCBA datasets at the top 1% and top 0.1% thresholds. With respect to docking tools, GNINA and SMINA dominated the top workflows on DEKOIS, with the QVINA variants rarely represented. On DUD-E, GNINA remained the most frequent engine, but PLANTS was also strongly represented, particularly at the top 0.1% threshold. This shift continued in the Lit-PCBA dataset, where PLANTS dominated the top workflows while GNINA was largely absent at the stringent top 0.1% threshold, suggesting dataset-dependent variability in docking tool effectiveness.

The observed dataset-dependent performance of docking methods is particularly intriguing. This distinction may reflect fundamental differences in the nature of these benchmarks. The DEKOIS and DUD-E datasets employ synthetic decoys designed to match physicochemical properties of active compounds [34, 35], whereas Lit-PCBA incorporates experimentally tested inactive compounds from real screening campaigns [36]. The performance advantage of PLANTS on Lit-PCBA is noteworthy, though it is important to emphasize that our current analysis focuses exclusively on final enrichment performance. While enrichment metrics are valuable indicators of screening effectiveness, they do not directly assess the ability of docking engines to recover correct binding poses, which is another critical aspect of SBVS quality [43]. A comprehensive comparison of docking engines should ideally incorporate both enrichment performance and pose prediction accuracy. We address this directly in section Binding-pose reproduction and Appendix G, where a dedicated cognate re-docking experiment on the CASF-2016 core set characterizes the pose-reproduction accuracy of DockM8’s docking engines and pose-selection scoring functions, showing that DockM8 reproduces native binding poses at rates consistent with established docking tools, with its pose-selection layer further improving near-native pose recovery.

Regarding pose-selection strategies, the deep-learning GenScore (across its variants) and RTMScore were the most frequently represented methods among top-performing workflows on the DEKOIS and DUD-E datasets, followed by KORP-PL and the CNN-based methods. A distinct shift was observed in the Lit-PCBA dataset, where KORP-PL and CNN-Score were the most frequent pose-selection methods, with PLANTS as the dominant docking engine. This divergence further underscores the unique challenges presented by the Lit-PCBA dataset and suggests that different scoring approaches may be better suited to different types of virtual screening problems.

#### Consensus and number of SFs

**Fig. 7:**
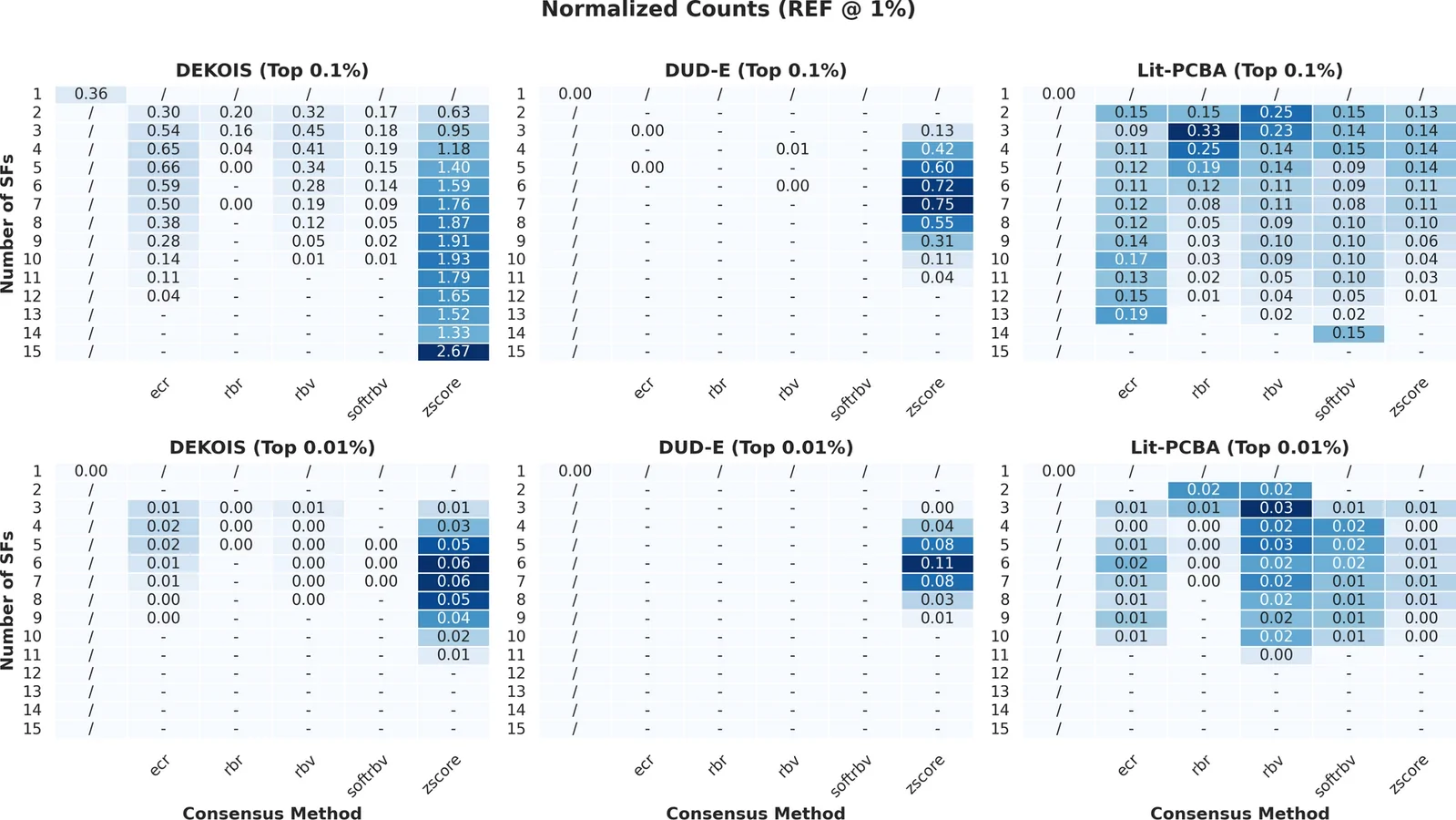
Success rate of DockM8 consensus methods and number of scoring functions at top performance thresholds (0.1%, 0.01%) on the DEKOIS, DUD-E, and Lit-PCBA datasets. Each cell shows the percentage of workflows with a specific combination of consensus method and number of scoring functions that reached the respective performance threshold, normalized against the total number of workflows with that same combination

Next, we evaluated how different consensus strategies and the number of SFs used in DockM8 workflows influence performance. To account for the uneven distribution of SF combinations, the heatmap in Fig. 7 reports normalized success rates rather than raw counts. Specifically, for each cell (representing a unique combination of consensus method and number of SFs), we computed the proportion of workflows that met the performance threshold, normalized by the total number of workflows using that combination. This normalization enables fair comparison across combinations with varying representation in the total workflow pool, allowing robust identification of consistently effective combinations regardless of their frequency of occurrence.

Figure 7 shows that the zscore method outperforms the other consensus methods on both the DUD-E and DEKOIS datasets at both thresholds. This trend was particularly pronounced in the DUD-E dataset, where at the top 0.1% and top 0.01% thresholds, workflows employing other consensus methods were nearly absent. In contrast, while zscore remained dominant in the DEKOIS dataset, other methods retained some presence at higher performance levels. For the Lit-PCBA dataset, by contrast, no single consensus method dominated. At the top 0.1% threshold, rbr was strongest at low numbers of scoring functions (peaking at three SFs), whereas ecr was the most consistent, maintaining a comparable success rate across a broad range of roughly two to thirteen SFs. At the more stringent top 0.01% threshold, none of the consensus methods showed a clear preference.

The varying performance of consensus methods across datasets reveals another layer of complexity in optimizing SBVS workflows. This pattern suggests that the normalization approach employed by zscore may be particularly effective for distinguishing active compounds from synthetic decoys; on the more challenging, experimentally derived Lit-PCBA dataset, by contrast, no single consensus strategy held a comparable advantage.

When looking at the relative frequency of the number of SFs included in the consensus, some interesting trends are visible. In DEKOIS at the top 0.1% and top 0.01% thresholds, we see that a number of SFs between 3–8 is more common regardless of the consensus method. A similar trend was observed for the DUD-E dataset. Again, for Lit-PCBA we observed different trends, with the preferred number of SFs depending on the consensus method. The rank-based rbr and rbv methods favored smaller sets of roughly two to seven SFs, whereas ecr remained effective across a considerably wider range, extending to larger sets.

This pattern suggests that the relationship between consensus performance and the number of SFs is not strictly linear. As the number of SFs increases, reaching effective consensus may become more challenging, particularly for datasets like Lit-PCBA, containing experimentally confirmed inactives that may differ from actives in more subtle ways. Different SFs may capture distinct aspects of ligand binding, and including too many functions might reduce effectiveness when these functions disagree on the ranking of challenging compounds.

Based on these findings, we recommend that users select consensus methods and SFs based on the nature of their screening problem. For targets with well-established binding modes and decoy sets, the zscore consensus method with a diverse set of 3–8 SFs, particularly including machine-learning scoring functions (MLSFs), may yield optimal results. For more challenging targets with realistic, experimentally derived screening data (as in Lit-PCBA), no single consensus method clearly outperformed the others; our results nonetheless indicate that combining between two and seven SFs, including knowledge-based approaches such as KORP-PL, is a sensible default.

#### Scoring function analysis

**Fig. 8:**
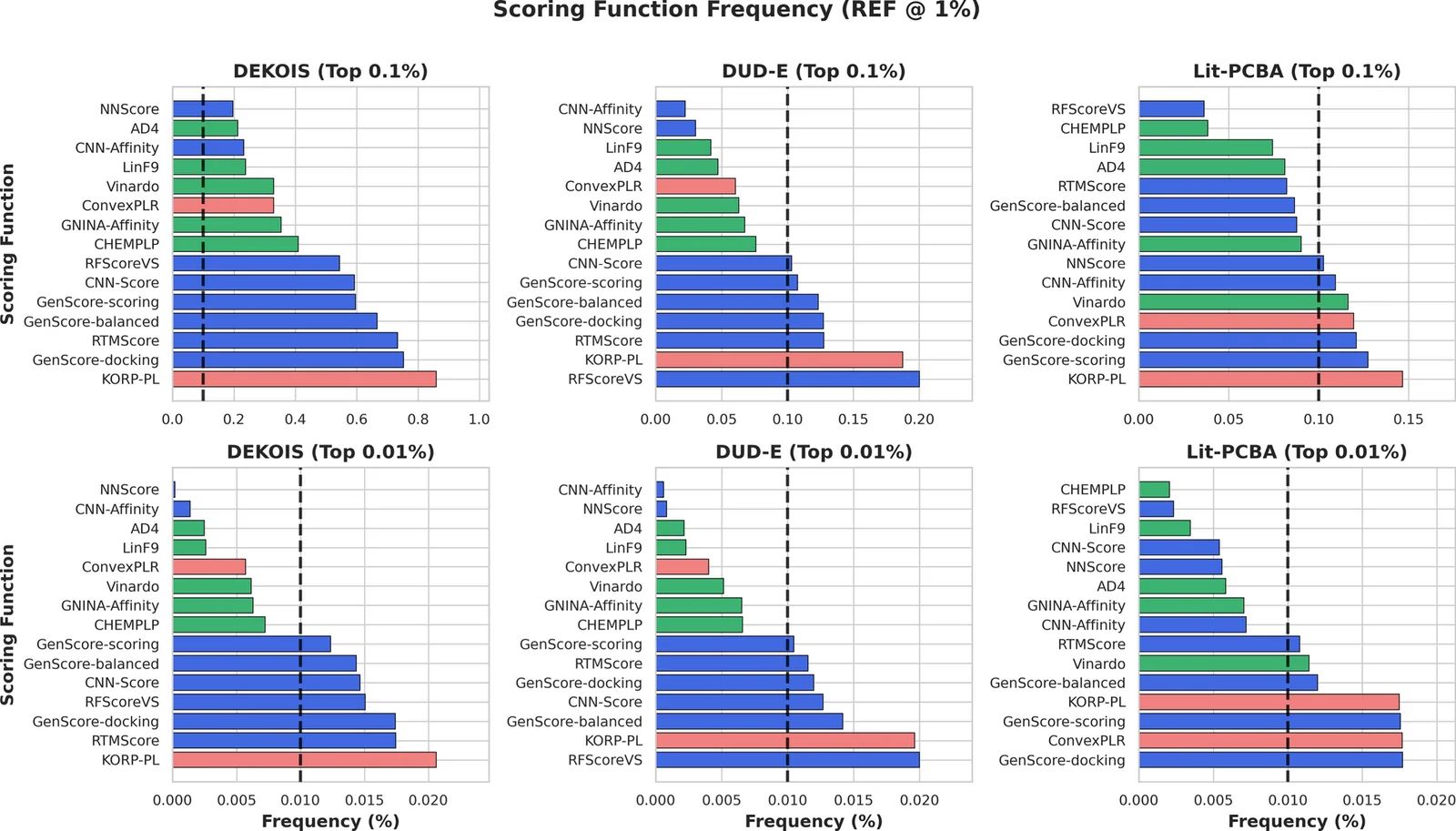
Enrichment of scoring functions in top-performing workflows at different performance thresholds (0.1%, 0.01%) across the DEKOIS, DUD-E, and Lit-PCBA datasets. Each bar represents the percentage of workflows containing a specific scoring function that reached the respective performance threshold, normalized by the total number of workflows containing that scoring function. The dashed vertical line indicates the expected baseline frequency (0.1%, or 0.01%). Bars are colored by scoring function category: machine learning (blue), empirical (green), and knowledge-based (red)

To gauge the relative performance and contributions of the various SFs, we performed a frequency analysis, which is shown in Fig. 8. This analysis quantifies how often each SF appears in top-performing workflows compared to its overall occurrence rate in the total workflow pool. We calculated the percentage of workflows containing a certain SF that reached the performance threshold, then compared this to the baseline expectation (shown as a vertical dashed line). SFs where bars extending beyond this baseline are enriched in top-performing workflows, indicating better than random performance.

Several interesting patterns emerge from this analysis. On the DEKOIS dataset, the knowledge-based KORP-PL together with MLSFs such as GenScore, RTMScore, and CNN-Score showed the highest enrichment across thresholds. The DUD-E dataset showed a closely similar pattern, with the same deep-learning functions (GenScore, RTMScore, and CNN-Score) alongside the knowledge-based KORP-PL again among the most enriched. The main difference was RF-Score-VS, which showed comparable, leading enrichment to KORP-PL at the more stringent thresholds. As we noted previously, the substantial enrichment of RF-Score-VS in DUD-E top workflows should be interpreted with caution, as this SF was developed using DUD-E data in its training set, making its strong performance on this particular dataset expected. On the Lit-PCBA dataset, ConvexPLR, GenScore, and KORP-PL showed the highest enrichment, with no clear trend among the remaining functions or by scoring-function category. These observations suggest that the relative advantages of different SF classes are context-dependent rather than cleanly split by category, underscoring the value of evaluating multiple scoring functions per target.

It is worth noting that in the case of DEKOIS at the top 0.01% threshold, all SFs show some degree of enrichment beyond the baseline. This reflects that nearly all tested SFs provide some advantage to performance. However, the key insight comes from examining the relative differences in enrichment between SFs, with those showing higher enrichment demonstrating stronger contributions to top performance.

### Binding-pose reproduction

Beyond screening (enrichment) power, the ability to reproduce the experimentally observed binding pose is a complementary measure of docking quality. Because the actives of the screening benchmarks used above lack experimentally determined bound poses, we assessed pose reproduction separately on the CASF-2016 core set (285 protein–ligand crystal structures) through cognate re-docking with all five DockM8 docking engines, selecting the top pose both by each engine’s native score and by each of the sixteen SFs (full protocol, per-engine results, and the complete pose-selector matrix in Appendix G). GNINA (49.5%) and SMINA (47.3%) most often ranked a near-native pose (≤ 2 Å) first, followed by PLANTS (43.3%); all docking engines sampled a near-native pose more often (63–76%) than their native score ranked it first, motivating DockM8’s dedicated pose-selection step. Used as pose selectors, the deep-learning RTMScore and GenScore recovered near-native poses most often (up to ${\approx }\,64\%$), followed by the knowledge-based KORP-PL and ConvexPLR (up to ${\approx }\,56\%$ and ${\approx }\,52\%$, respectively), confirming that the pose-selection layer improves near-native pose recovery beyond the engines’ native ranking. Overall, DockM8 reproduces native binding poses at rates consistent with established docking tools on this benchmark [44].

### User-friendly open-source tools

In medicinal chemistry, using VS software is crucial but challenging. Open-source tools often require programming knowledge and are rarely updated, limiting their adoption by chemists with limited computational skills. Commercial tools, while user-friendly, are expensive and inaccessible to smaller academic groups. Our analysis shows that commercial tools do not necessarily perform better on our datasets. To address these issues, we introduce DockM8, a user-friendly framework that requires minimal programming expertise and uses open-source components. This approach makes SBVS more accessible and challenges the idea that higher cost equates to higher quality. DockM8 aims to provide an easy-to-use SBVS workflow with extensive documentation for operation and installation on various platforms. It includes a basic GUI to minimize coding and command-line usage.

### Limitations and future directions

While DockM8 demonstrates strong performance advantages, opportunities for further refinement and expansion are present. Our rigorous retrospective analysis on multiple benchmark datasets provides strong evidence for the effectiveness of target-optimized workflows in virtual screening. The AVE analysis shows that the generalizability of optimized workflows is strongly target-dependent so prospective validation studies will be important to establish DockM8’s utility in real-world drug discovery campaigns.

A further methodological caveat concerns structure selection: our use of the highest-resolution crystal structure available for each Lit-PCBA target, while consistent with SBVS best practices, may have contributed to DockM8’s strong performance on that dataset. Prior work has shown that scoring performance can vary appreciably with the specific crystal structure employed [45]. Future work will therefore assess the robustness of DockM8 workflows across a range of structural resolutions to disentangle this factor from the platform’s intrinsic strengths.

A related limitation concerns receptor flexibility: the current workflow performs rigid-receptor docking, so induced-fit effects and cryptic conformational changes upon ligand binding are not modeled. As with ensemble docking, explicitly accounting for such receptor flexibility could improve the discrimination of actives from inactives when the bound conformation differs substantially from the chosen structure, albeit at the cost of an enlarged search space.

The current implementation of DockM8 successfully leverages established open-source SFs, with noteworthy performance from empirical and knowledge-based approaches. Expanding our SF repertoire with additional state-of-the-art MLSFs and developing targeted consensus methodologies presents exciting possibilities for further performance enhancement. In addition, although DockM8’s default configuration is intentionally fully open-source, its modular architecture allows further engines and SFs (including widely used commercial tools such as Glide [7]) to be integrated; supporting such options is a natural extension that would broaden adoption in industrial settings.

The computational demands of comprehensive consensus approaches represent a practical consideration when screening exceptionally large compound libraries. Significant speed improvements could be achieved by transitioning from multiple .sdf files to a database system [46] and incorporating accelerated docking methods like Karmadock [47] or UMol [48]. Integration of sophisticated pre-filtering techniques and active-learning approaches such as HASTEN [49] and DeepDocking [50] could enable rapid SBVS of extensive libraries. Enhanced docking pose and consensus result visualization capabilities in the GUI are also planned. We enthusiastically invite the computational chemistry community to contribute to DockM8’s ongoing development via our GitHub repository (https://github.com/DrugBud-Suite/DockM8).

### Usage recommendation

The configurability of DockM8 across pose-selection, consensus, and scoring methods enables highly tailored VS workflows. Based on our benchmarking results (section Evaluation of component performance across datasets), we provide the following practical recommendations as a starting point when biologically validated active compounds are unavailable:

- Consensus: On the Lit-PCBA dataset, no single consensus method clearly outperformed the others, but ecr gave the most consistent performance across scoring-function set sizes. By rewarding compounds ranked highly by *any* scoring function (a conditional “or”), ECR can recover actives missed by individual functions, which may explain its robustness on this more challenging dataset. Although further validation in real-world scenarios is necessary, it is likely to be a good starting point.
- Docking selection: While our analysis focused primarily on enrichment potential, we recognize that docking programs offer additional benefits beyond enrichment (such as pose RMSD accuracy and protein-ligand interaction recovery). For computationally efficient screening, we recommend PLANTS as it offers a good balance of speed and performance. For scenarios where computational resources are less constrained, GNINA is recommended as it has demonstrated superior performance both in our analysis and in other studies [3].
- Pose-selection: The deep-learning GenScore and RTMScore, together with CNN-Score, were the most frequently selected pose-selection methods in top-performing workflows on DEKOIS and DUD-E; on Lit-PCBA, KORP-PL and CNN-Score were most effective. GNINA-Affinity remains a robust, computationally efficient option.
- SFs: Our analysis suggests that using 2–6 SFs provides an optimal balance between performance and computational cost. KORP-PL and ConvexPLR offer excellent performance with minimal computational overhead. Vinardo and GNINA-Affinity also perform well across diverse targets. Among ML approaches, GenScore (scoring and docking variants) and RTMScore showed the strongest enrichment on DEKOIS and DUD-E, with CNN-Score and CNN-Affinity also performing well; GenScore additionally remained competitive on Lit-PCBA. Whenever available, known well-performing SFs on the target at hand should be included in the consensus.

## Conclusions

In this work, we introduced an open-source consensus docking workflow DockM8. We presented our comprehensive evaluation of DockM8, using three datasets: DEKOIS 2.0, DUD-E, and Lit-PCBA. We demonstrated its competitive performance in SBVS over existing methods, including state-of-the-art ML and advanced consensus approaches. The versatility and adaptability of DockM8, characterized by its ability to handle a variety of docking algorithms and SFs, enable it to perform competitively across a range of targets; our generalizability analysis (section Generalizability assessment and predictive capability of DockM8 workflows) nonetheless shows that the extent to which an optimized workflow transfers to new data is target-dependent.

Furthermore, DockM8 addresses critical challenges in the field, particularly the accessibility and usability of SBVS tools in academic research. Its user-friendly interface, minimal programming requirements, and open-source nature democratize the use of advanced SBVS methods. This makes computational drug discovery tools accessible to researchers without specialised computational infrastructure, demonstrating that competitive SBVS performance is achievable with entirely open-source components.

Despite its current strengths, there remain opportunities for further enhancement of the DockM8 workflow. Areas such as the speed of the procedure, integration of advanced machine-learning docking and active-learning algorithms, and improvements to the GUI are identified as key directions for future development. The potential inclusion of pre-filtering steps and the combination of DockM8 with active learning methodologies could further augment DockM8’s efficacy and efficiency in handling large compound libraries.

Given these findings, we invite the computational and medicinal chemistry communities to participate in the ongoing development of DockM8. Its open-source nature and our commitment to collaboration ensure continuous innovation in virtual drug screening. By leveraging collective expertise, we aim to make DockM8 an even more powerful tool, significantly advancing the field.

## Methods

The DockM8 pipeline consists of seven steps, automating the entire process from data pre-processing, followed by docking and scoring, and consensus ranking, as described in sections Ligand library preparation through Consensus methodologies. DockM8 is available at https://github.com/DrugBud-Suite/DockM8.

### Main workflow

#### Ligand library preparation

Ligands are supplied to DockM8 as an .sdf file. The ligand library is first standardized using the ChEMBL-structure-pipeline [51] library, which uses RDKit [52] to check, standardize and desalt ligands. We selected Gypsum-DL for compound protonation [53] as it is open-source and has demonstrated strong performance in this task. Additionally, since certain docking algorithms require pre-generated 3D structures as input, Gypsum-DL is also used to generate a single 3D conformer for each ligand. By default, DockM8 enumerates protonation states within a pH window of 6.4 to 8.4.

#### Receptor preparation

DockM8 offers comprehensive protein structure preparation through a modular pipeline that handles various input formats. Users can provide structures as Protein Data Bank (PDB) codes, UniProt accession numbers, or local PDB files. For PDB codes, structures are fetched directly from the RCSB PDB database [54], while UniProt codes trigger retrieval of AlphaFold-predicted structures [55]. The preparation process includes options for fixing nonstandard residues, addressing missing residues, and managing heteroatoms and water molecules (powered by PDBFixer [56]). For protonation, DockM8 provides two methods: PDBFixer with customizable pH values (default 7.0), or the Protoss program. Protoss optimizes the hydrogen bonding network in protein–ligand complexes, addressing the complexities of hydrogen bonding, tautomerism, and ionization to enable precise analysis of binding modes and calculation of associated binding energies [57]. This provides streamlined access to ready-to-dock protein structures. The protonation method can be explicitly specified as "protoss" or "pdbfixer" (with a default pH of 7.0 for PDBFixer). Users can configure these preparation parameters according to their specific requirements, with sensible defaults ensuring accessibility for novice users. For cases requiring specialized protonation states not handled by the automated pipeline, users can pre-process their protein input files using external tools (e.g. PyMOL [27]) and disable automatic protonation in DockM8.

#### Pocket detection

Users are presented with multiple strategies for binding site selection. The Reference and radius of gyration (RoG) options make use of a co-crystallized ligand, which needs to be supplied as a separate file. Alternatively, an already docked pose could also be used. In the Reference method, DockM8 automatically determines the ligand’s center of mass (CoM) and defines a box (the dimensions of which can be specified by the user) around this CoM. The RoG method determines the box’s center coordinates in the same way as before but uses the previously defined reference ligand’s RoG to define the box size. This was shown to be a suitable method to determine box size for AutoDock Vina [58].

Alternatively, if no crystal ligand is available, DockM8 can make use of the DoGSiteScorer algorithm which combines pocket finding along with pocket characterization to select druggable binding sites [59]. DockM8’s query of DoGSiteScorer via the proteins.plus webserver [60] outputs the largest-volume binding site by default. An option to select binding sites based on other metrics provided by DogSiteScorer is also included (available metrics are: Volume, Surface, Depth and Druggability Score).

Finally, the user can supply custom box center and size values, both in the GUI and by command line. The binding site definition is then used for all further docking and rescoring.

#### Docking

DockM8 currently supports five distinct docking algorithms: SMINA [21], GNINA [3], QVINA-W [61], QVINA2 [62], and PLANTS [63]. These algorithms represent a range of approaches, from improved versions of AutoDock Vina to convolutional neural network-based methods and ant colony optimization. Detailed descriptions of each algorithm are provided in Appendix B.2. The user can choose from any combination of these docking algorithms integrated in DockM8.

Docking calculations are parallelized across available CPU cores for efficiency. After docking, poses can be filtered using the PoseBusters [64] library, which implements pose quality checks using RDKit. We developed a custom configuration file (posebusters_config.yml) to reduce calculation times while retaining critical quality checks, which can be adjusted by the user.

#### Pose-selection

Pose-selection is a standard step in molecular docking workflows, where the most promising binding poses are identified from the initial set of generated conformations. In DockM8, this step is particularly important for consensus scoring, as evaluating all generated poses with multiple SFs would be computationally prohibitive. By selecting the best-scoring pose for each ligand at this stage, the number of poses carried forward is substantially reduced, enabling efficient downstream rescoring with diverse SFs. Furthermore, decoupling pose-selection from final ranking allows users to employ different SFs for each task, which is advantageous since SFs that excel at identifying near-native poses do not necessarily perform well at ranking compounds by binding affinity, and vice versa [44]. Any of the 17 SFs supported by DockM8 can be used for pose-selection, with the top-ranked pose for each ligand retained for further calculations.

#### Rescoring

DockM8 is bundled with 17 different SFs, which include empirical, semi-empirical, knowledge-based, and machine learning categories. Each SF is characterized by its score range and optimization direction (whether lower or higher values indicate better binding). Users can select any combination of these SFs during rescoring; the selected poses are evaluated by each function independently, producing a score matrix where rows represent selected ligand poses (one per molecule, following pose-selection) and columns represent the individual SF scores. This score matrix serves as input for the subsequent consensus calculation. As mentioned previously, any of these SFs can also be used to select docking poses. A list and description of the SFs available in DockM8 is shown in Table 2.

**Table 2:** List of scoring functions available in DockM8

SF	Type	Note
AutoDock4 [4]	Semi-empirical	SF from AutoDock4
GNINA-Affinity [3]	Empirical	Empirical SF (GNINA)
LinF9 [65]	Empirical	Improved scoring based on 9 empirical terms
PLP [63]	Empirical	Piecewise linear potential (4 terms)
CHEMPLP [63]	Empirical	PLP+GOLD terms for metals and torsions
Vinardo [66]	Empirical	Improved empirical scoring based on Vina
KORP-PL [67]	Knowledge-based	Coarse-grained knowledge-based scoring
ConvexPLR [68, 69]	Knowledge-based	SF incorporating entropic terms
CNN-Score [3]	Machine-learning	CNN pose scoring model (GNINA)
CNN-Affinity [3]	Machine-learning	CNN binding affinity prediction (GNINA)
RFScoreVS [70]	Machine-learning	Random Forest-based SF
RTMScore [11]	Machine-learning	Graph transformer-based SF
GenScore-scoring [38]	Machine-learning	Extension of RTMScore (GatedGCN_ft_1.0)
GenScore-docking [38]	Machine-learning	Extension of RTMScore (GT_0.0)
GenScore-balanced [38]	Machine-learning	Extension of RTMScore (GT_ft_0.5)
SCORCH [71]	Machine-learning	Consensus of 3 ML models
NNScore [72]	Machine-learning	Neural network-based affinity prediction

CNN: convolutional neural network

#### Consensus methodologies

DockM8 currently supports five different consensus methods, combining traditional and modern strategies to give users flexibility in adapting the protocol to their target. Before applying any consensus method, all SF scores undergo a standardization procedure. Scores are normalized to the [0, 1] range using min-max scaling based on the observed minimum and maximum values within each SF for the current dataset. The direction is then harmonized such that a value of 1 always represents the best score, regardless of whether the original SF favors lower or higher values; for SFs where lower scores indicate better binding, the normalized values are inverted (see Appendix C.4). This data-driven standardization ensures that all consensus methods operate on comparable, direction-consistent inputs without imposing arbitrary theoretical bounds.

Z-score: The score (*s*) assigned to a molecule is normalized within each SF by applying Z-score standardization, using the mean (μ) and standard deviation (σ) of all scores in that SF (Eq. 1) [73]. Let *n* be the total number of SFs, $s_y^{x}$ the score of molecule *x* for SF *y*, $\mu _{y}$ the average score and $\sigma _{y}$ the standard deviation of all scores of this SF; then:

1 $$\begin{aligned} \text {Z-score}_x = \frac{1}{n} \sum \limits _{y} \frac{s_{y}^{x} - \mu _{y}}{\sigma _{y}}, \end{aligned}$$

Rank by Rank (RbR): The candidate molecules are selected based on the rank for all the selected SFs (Eq. 2) [16]. Let $r_y^{x}$ be the rank of the molecule *x* for SF *y* and *n* be the total number of SFs; then the output of the RbR consensus is given by:

2 $$\begin{aligned} {RbR}_x = \frac{1}{n} \sum \limits _{y} r_y^{x}, \end{aligned}$$

Our implementation assigns higher ranks to molecules with higher original scores and subsequently inverts the mean rank, ensuring that all consensus scores follow a higher-is-better convention for ease of use and straightforward comparison across methods.

Rank by Vote (RbV): Molecules receive votes if their normalized score exceeds a per-SF threshold set at the 95th percentile, with the total score being the sum of votes across all SFs [16]. Let $s_y^{x}$ be the normalized score of molecule *x* for SF *y*, $t^y$ the 95th-percentile threshold of the normalized scores for SF *y*, and *n* the total number of SFs; then:

3 $$\begin{aligned} {RbV}_x = \sum \limits _{y} 1\!\!1(s_y^{x} > t^y) + \epsilon _x, \end{aligned}$$

where $1\!\!1(\cdot )$ is the indicator function returning 1 if the condition is satisfied and 0 otherwise. The term $\epsilon _x \in [0, 0.2)$ is a Z-score-based tiebreaker computed from the standardized scores, added as a fractional component to ensure deterministic rankings when molecules receive the same number of votes.

Soft Rank by Vote: (Soft RbV) This enhancement of RbV provides more nuanced rankings using continuous vote values between 0 and 1 based on a sigmoid function (Eq. 4). Let $s_y^{x}$ be the score of molecule *x* for SF *y*, $t^y$ the threshold (95th percentile), $\sigma ^y$ the standard deviation, and σ a steepness parameter; then:

4 $$\begin{aligned} {SoftRbV}_x = \sum \limits _{y} \frac{1}{1 + \exp (-\frac{s_y^{x} - t^y}{\sigma ^y \cdot \sigma })}, \end{aligned}$$

This creates a smooth transition around the threshold, with σ controlling the steepness. The default value of σ=0.1 was chosen empirically to concentrate most of the vote transition close to the threshold, preserving the selective character of RbV while providing sufficient smoothing to differentiate molecules near the decision boundary and eliminate tie-breaking issues.

Exponential Consensus Ranking (ECR): The ECR method of Palacio-Rodríguez et al. [74] assigns an exponential score to each molecule based on its rank from each SF, then sums these contributions (Eq. 5). Let $r_y^{x}$ be the rank of molecule *x* for SF *y* and σ the expected value of the exponential distribution; then:

5 $$\begin{aligned} {ECR}_x = \frac{1}{\sigma } \sum \limits _{y}\exp ({-\frac{r_y^{x}}{\sigma }}), \end{aligned}$$

The parameter σ establishes the effective number of top-ranked molecules considered from each SF. In our implementation, σ is set to 5% of the total molecules to match the original implementation by Palacio-Rodríguez et al. [74]. A key advantage of ECR is that molecules well-ranked by *any* SF receive high scores, functioning as a conditional “or” rather than the conditional “and” implicit in traditional intersection-based consensus approaches. This enables recovery of active compounds that may be poorly ranked by one SF but highly ranked by others. Furthermore, ECR results remain largely independent of the σ parameter over a wide range [74].

### Additional features

#### Single and ensemble docking modes

In order to partially account for protein flexibility, DockM8 supports two major operational modes:

Single Docking Mode: This mode performs "traditional" molecular docking against a single protein structure. In this case, the output of the workflow is a list of compounds ranked by their consensus score.

Ensemble Docking Mode: In this mode, users can select multiple protein structures for the docking process. Each structure is processed sequentially, following the same protocol as single docking mode. Users define a percentage threshold (e.g., 1% or 5%) that specifies which fraction of the top-ranked compounds to consider. For each protein conformation, compounds are ranked by their consensus score and those within the specified top percentage are identified. The final output contains only compounds that rank within this top percentage across *all* protein conformations simultaneously. For example, with a 5% threshold and three protein structures, only compounds that score in the top 5% for every structure are retained in the final selection.

#### Performance evaluation

A comprehensive suite of virtual screening metrics was implemented to evaluate DockM8 workflow performance (see Appendix C for mathematical formulations).

These metrics comprise two complementary categories. Threshold-dependent metrics quantify performance at specific rank cutoffs, addressing the practical question of active compound recovery within a defined selection fraction. This category includes enrichment measures (Enrichment Factor (EF), REF, Power Metric (PM), ROC Enrichment (ROCE)) that quantify fold-improvement over random selection, and classification quality measures (Correct Classification Rate (CCR), Matthews Correlation Coefficient (MCC), Cohen s Kappa Coefficient (CKC)) that account for both sensitivity and specificity.

Threshold-independent metrics evaluate discrimination across the complete ranked list without requiring fixed cutoffs. AUC-ROC and Area Under the Precision-Recall Curve (AUPR) summarize overall ranking quality by integrating performance across all possible thresholds, while BEDROC and Robust Initial Enhancement (RIE) emphasize early recognition of actives, a property of particular relevance for virtual screening applications where only top-ranked candidates proceed to experimental validation. The AUC-ROC and AUPR metrics are calculated using the sklearn library, while BEDROC and RIE are implemented using the rdkit.Scoring module.

Prior to analysis, all SFs are normalized to a common scale (0–1), with 1 representing optimal scores regardless of the native scoring convention. See section Consensus methodologies.

#### Decoy generation

Due to the considerable number of possible conditions under which DockM8 can be run, the user needs to be able to determine which choices are most appropriate for the target under study. One of the ways to achieve this is to generate decoy molecules that closely resemble the physicochemical properties of known active compounds for that particular target. If the screening software can discern the actives from the decoys effectively, the chances of being able to discern potentially active compounds from a real docking library are increased. When decoy generation is activated and a list of known active compounds is supplied, DockM8 will make use of the DeepCoy [75] library to generate a user-specified number of decoys.

Subsequently, the user can choose which conditions (docking, pose-selection, and rescoring) are to be explored. This allows the user to limit the search space of conditions if desired. DockM8 will then run the workflow using every possible combination of the supplied conditions to determine which one is best able to distinguish active compounds from decoy molecules. The optimum conditions will then be reported and be automatically used for the screening of the library of interest.

### Implementation

The DockM8 implementation is designed to be modular and user-friendly, with both command-line and graphical interfaces available. All source code is openly accessible via the GitHub repository: https://github.com/DrugBud-Suite/DockM8.

#### Graphical user interface

**Fig. 9:**
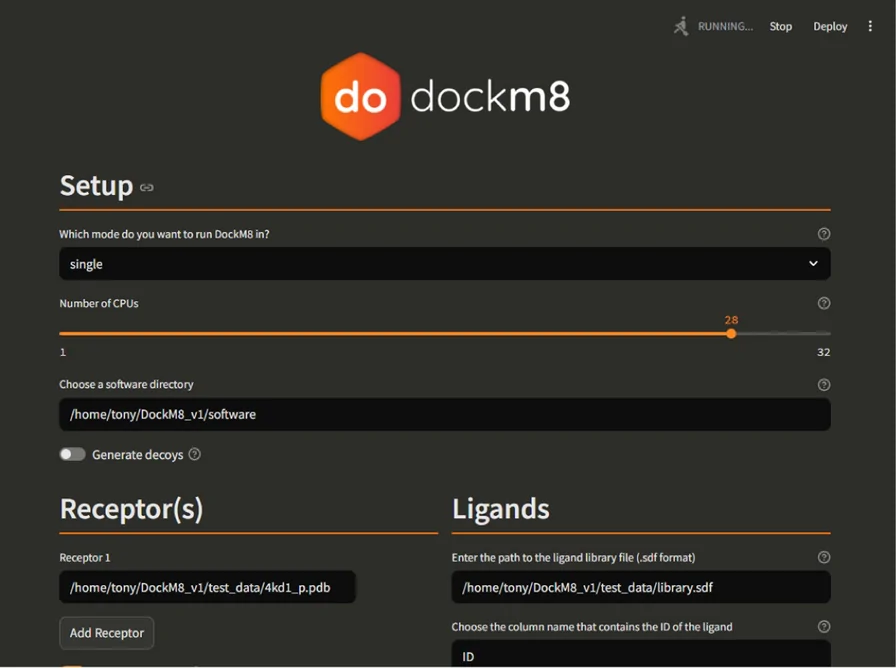
The DockM8 graphical user interface. Screenshot of the Streamlit-based interface for configuring molecular docking simulation inputs

A simple GUI is provided for DockM8 via the Streamlit framework [76]. If the streamlit library is installed, the GUI can be launched by the command: streamlit run gui.py. The interface is shown in Fig. 9. The GUI enables users to configure all aspects of a docking workflow, including receptor and ligand preparation, pocket detection method, docking programs, scoring functions, and consensus algorithms. Users can also toggle between single and ensemble docking modes, optionally generate decoys for benchmarking, and monitor the docking progress through a live log output.

#### Technical details

The DockM8 workflow (Fig. 1) was developed for Python 3.10 and the Ubuntu (version 20 and above) operating system, although it can be run in Windows Subsystem for Linux version 2 if required. Multiprocessing capabilities are integrated into DockM8, particularly during the docking and rescoring phases as these are the most resource-intensive. Allocation of individual processing runs across available CPU cores is managed via Python’s multiprocessing facilities.

### Benchmark datasets and strategy

To evaluate DockM8’s performance across diverse virtual screening scenarios, we selected three widely used benchmark datasets. Each dataset was processed using a standardized workflow, with specific adaptations detailed in the subsections below. DockM8 v1.1.1 was used for all benchmarking efforts.

#### DEKOIS 2.0

The DEKOIS 2.0 library (81 targets) was downloaded via the link provided in the original publication [35]. Two targets were omitted due to unexpected difficulties with QVINA2 docking, leaving 79 targets (see Appendix D). The reference ligand and protein files were used as is in the DockM8 workflow. The docking library was generated by combining the actives and decoys into one SDF file and labeling each with 0 (for decoys) or 1 (for actives) to allow for the calculation of the performance metrics. The DockM8 conditions used are described in Appendix F.

#### DUD-E

A subset (see Appendix D) of the DUD-E library (28 targets) was downloaded via the link provided in the original publication [34]. A representative portion of the dataset was selected to ensure tractable computation time and resource usage while maintaining statistical relevance. Dataset preparation was carried out in the same manner as for the DEKOIS dataset. The DockM8 conditions used are described in Appendix F.

#### Lit-PCBA

The Lit-PCBA library (15 targets) was downloaded via the link provided in the original publication [36]. OPRK1 was omitted to ensure tractable computation time, leaving 14 targets (see Appendix D). Dataset preparation was carried out in the same manner as for the DEKOIS dataset. Since the dataset provides multiple PDB structures for each target, we selected the structure with the highest resolution. The DockM8 conditions used are described in Appendix F.

#### Performance comparison methodology

To establish a comprehensive performance comparison between DockM8 and existing methodologies, we conducted an extensive literature review of publications that utilized the DUD-E, DEKOIS, or Lit-PCBA benchmark datasets. We systematically collected reported performance metrics from these studies (AUC-ROC, BEDROC, and EF at various thresholds). These collected metrics are provided in the Supplementary Information, along with a link to the publication from which they were taken.

Since most publications predominantly reported EF, we developed a computational pipeline to back-calculate additional performance metrics. The pipeline processes literature-reported EF values in conjunction with dataset-specific statistics to derive a comprehensive set of metrics. For each target-method pair, the algorithm computes the number of active compounds in the selection ($n_s$) based on the reported enrichment factors, the total number of compounds (N), the total number of actives (n), and the selection size ($N_s$).

The number of active compounds in the selection ($n_s$) is derived from the reported enrichment factor using:

6 $$\begin{aligned} n_s = \frac{\text {EF}}{100} \times N_s \end{aligned}$$

where EF denotes the percentage-based enrichment factor (i.e., the hit rate within the selection) and $N_s$ represents the number of compounds in the top-ranked selection at a given threshold.

Using these fundamental parameters, the pipeline calculates several metrics, including REF, PM, ROCE, CCR, MCC, and CKC. The mathematical formulations for these metrics are detailed in Appendix C. The Python script containing the pipeline is provided in the GitHub repository.

For our comparative analysis, we primarily utilize the REF, defined as the percentage of the maximum possible enrichment achieved by a method (at a certain selection threshold). This metric is particularly valuable as it normalizes enrichment relative to the theoretical maximum, providing a clearer indication of how effectively each method identifies active compounds. Unlike the raw enrichment factor, which inherently depends on the proportion of actives in the entire dataset (n/N), the REF removes this dependency by normalizing against the best achievable outcome at any given selection threshold, enabling meaningful comparisons across datasets with different active ratios.

Transparency in method comparison: In our comparative analysis, we have included (to the best of our knowledge) all published methods that reported performance on the DEKOIS, DUD-E, or Lit-PCBA benchmarks, regardless of potential methodological advantages or limitations. To ensure transparency and fair interpretation, methods with specific characteristics are clearly labeled in our performance plots:

Methods tested on only a subset of targets within a benchmark dataset are denoted with a † symbol, along with the fraction of targets tested (e.g., "EHIGN†(7/15)" indicates the method was tested on only 7 out of 15 Lit-PCBA targets). Methods that were partially or fully trained on a benchmark dataset they were evaluated on are marked with a ‡ symbol (e.g., "DeepScore‡" in the DUD-E plot indicates the method was trained using DUD-E data).

This comprehensive approach to reporting allows for a fair assessment of all available methods while acknowledging factors that might influence performance comparisons. Rather than excluding potentially valuable methods from our analysis, we provide readers with the necessary context to make informed interpretations of the reported performance metrics. For methods falling into these categories, we recommend cautious interpretation of their reported performance relative to other approaches tested on a more complete dataset.

### DockM8 workflow selection and generalization assessment

To evaluate the performance and generalizability of DockM8 workflows, we employed two complementary selection strategies: target-specific optimization and cross-target robustness. These approaches were further validated using asymmetric validation embedding analysis to ensure methodological rigor and minimize retrospective bias.

#### DockM8-best and DockM8-median selection methodology

The modular architecture of DockM8 enables extensive configurability across all workflow stages. With five docking algorithms, 17 scoring functions available for pose-selection, and any combination of these 17 SFs available for rescoring (yielding $2^{17} - 17 - 1 = 131{,}054$ possible SF combinations), along with five consensus methods, the platform can theoretically generate over 55 million distinct workflow configurations. This combinatorial explosion necessitates systematic approaches for workflow prioritization and selection. We therefore employed two distinct strategies that address complementary practical scenarios: target-specific optimization for known targets where retrospective analysis is feasible, and cross-target robustness for prospective screening where generalizability is paramount.

DockM8-best identifies target-optimized workflows by employing a sequential multi-criteria selection approach. For each target, workflows are strictly prioritized by sorting first on REF 1%, then REF 0.1%, followed by PM 1%, BEDROC, and finally AUC-ROC, with the best-performing configuration selected. This target-specific optimization ensures maximum enrichment for each individual target.

In contrast, DockM8-median identifies a single generalizable workflow across all targets by maximizing the median REF 1% value. The median was specifically chosen over the mean to prevent high-performing outliers from skewing the selection process. This approach selects workflows demonstrating robust performance across the entire target space rather than excelling on specific targets.

#### Asymmetric validation embedding analysis

To rigorously assess the generalization capabilities of DockM8 and address potential concerns regarding retrospective workflow selection, we conducted an analysis using the AVE partitioning of the Lit-PCBA dataset provided by the original authors [36]. The AVE methodology divides each target’s dataset into training and validation subsets using a sophisticated embedding approach that minimizes information leakage while maintaining chemical diversity.

For all 14 Lit-PCBA targets, we evaluated DockM8 workflow performance. To ensure computational tractability, this analysis focused on workflows utilizing only the SMINA docking engine. We compared the performance achieved on the AVE training set versus the AVE validation set.

Top-performing workflows were selected based on their BEDROC score (α=80.5) achieved on the AVE training set. BEDROC was chosen for workflow selection as it emphasizes early enrichment performance, which is a primary goal in practical VS campaigns. Specifically, we selected the top 0.1% of workflows according to this criterion.

To evaluate the correlation of performance between the training and validation sets, we utilized the AUC-ROC metric. AUC-ROC was selected for this evaluation due to its properties as a threshold-independent metric that provides a global assessment of ranking quality. Furthermore, unlike early enrichment metrics such as EF, AUC-ROC is less susceptible to quantization effects and statistical instability when dealing with low numbers of active compounds, as encountered in several of the AVE validation sets (see Table 7). Specifically, EF values are discrete and exhibit discontinuous jumps when recovering additional active compounds (e.g., a single extra active can shift EF from x to y without intermediate values), making correlation analysis difficult to interpret. In contrast, AUC-ROC provides continuous values that enable robust assessment of performance consistency across dataset splits. The analysis involved plotting the AUC-ROC achieved on the training set against the AUC-ROC achieved on the validation set for both all workflows and the top 0.1% best-performing workflows as ranked by BEDROC. This visualization demonstrates whether workflows identified as optimal on the training data maintain strong performance on the validation data, thereby validating that top-ranked workflows generalize effectively across different compound sets rather than being specific to the training targets.

## Supplementary Information

Supplementary material 1. Collected performance metrics from published studies evaluated on the DEKOIS 2.0 benchmark, with links to original publications

Supplementary material 2. Supplementary figures (Figures S1–S16) presenting additional performance comparisons using AUC-ROC, BEDROC, EF, PM, and REF metrics across the DEKOIS 2.0, DUD-E, and Lit-PCBA datasets, together with the complete AVE generalizability analysis for all 14 Lit-PCBA targets and its dependence on the target active-to-inactive ratio (Figure S16)

Supplementary material 3. Collected performance metrics from published studies evaluated on the DUD-E benchmark, with links to original publications

Supplementary material 4. Collected performance metrics from published studies evaluated on the Lit-PCBA benchmark, with links to original publications

## Figures and Tables

**Fig. 10:**
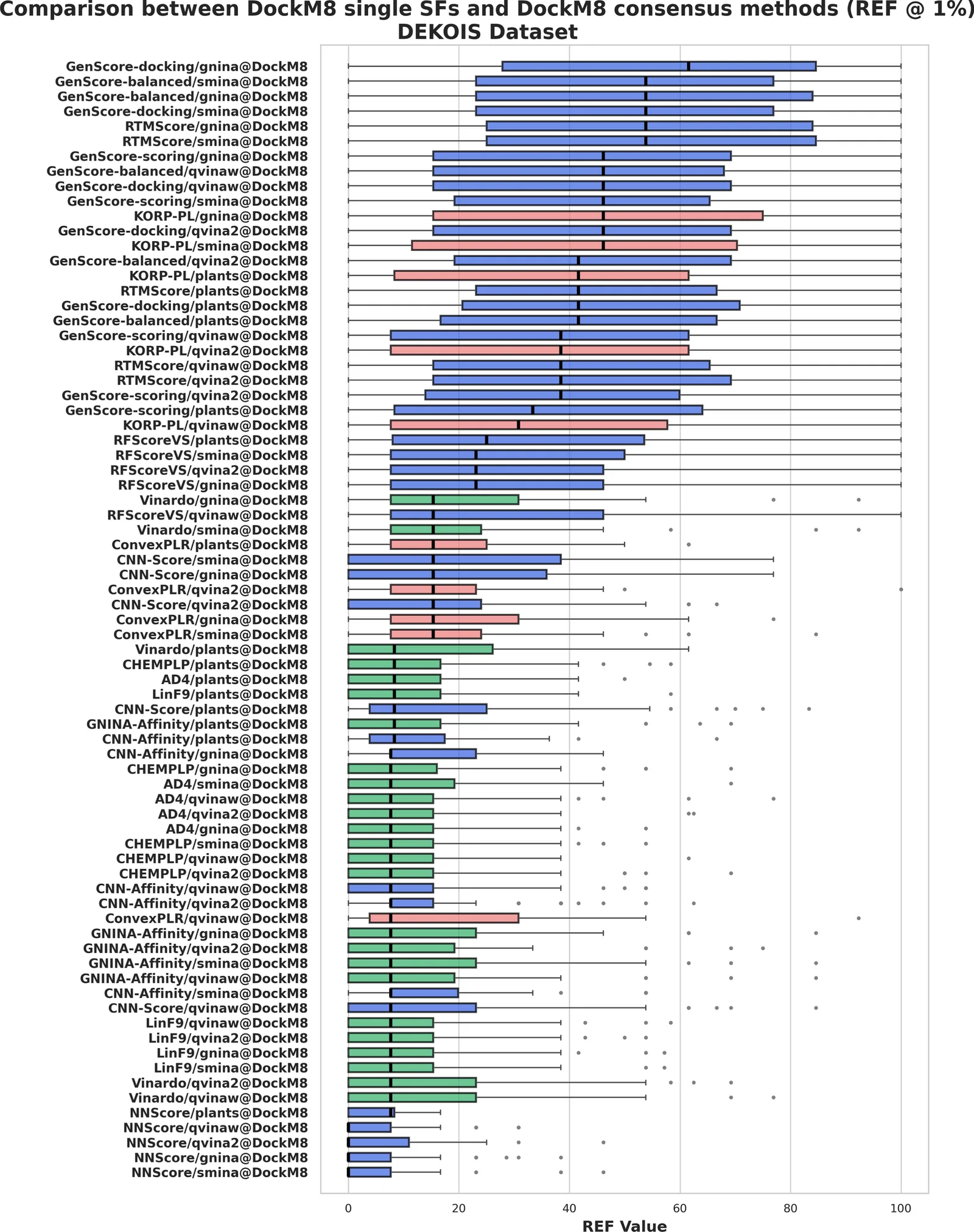
Single SFs vs. consensus methods on DEKOIS. Comparison by $\hbox {REF}_{1\%}$. Machine-learning scoring functionss are shown in blue, empirical scoring functions in green, knowledge-based scoring functions in light red, and consensus methods in orange

**Fig. 11:**
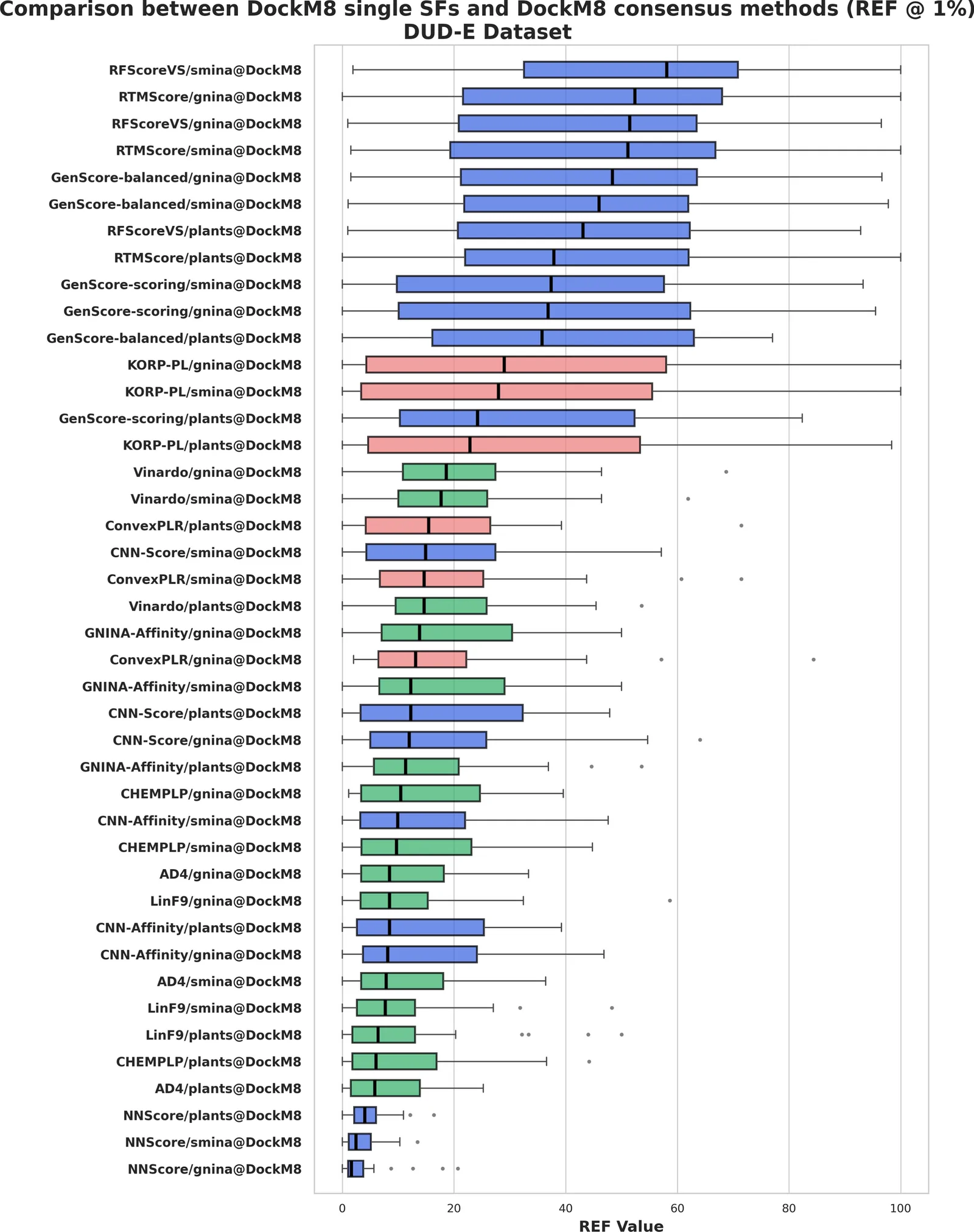
Single scoring functions vs consensus methods on DUD-E. Comparison by $\hbox {REF}_{1\%}$. Machine-learning scoring functions are shown in blue, empirical scoring functions in green, knowledge-based scoring functions in light red, and consensus methods in orange

**Fig. 12:**
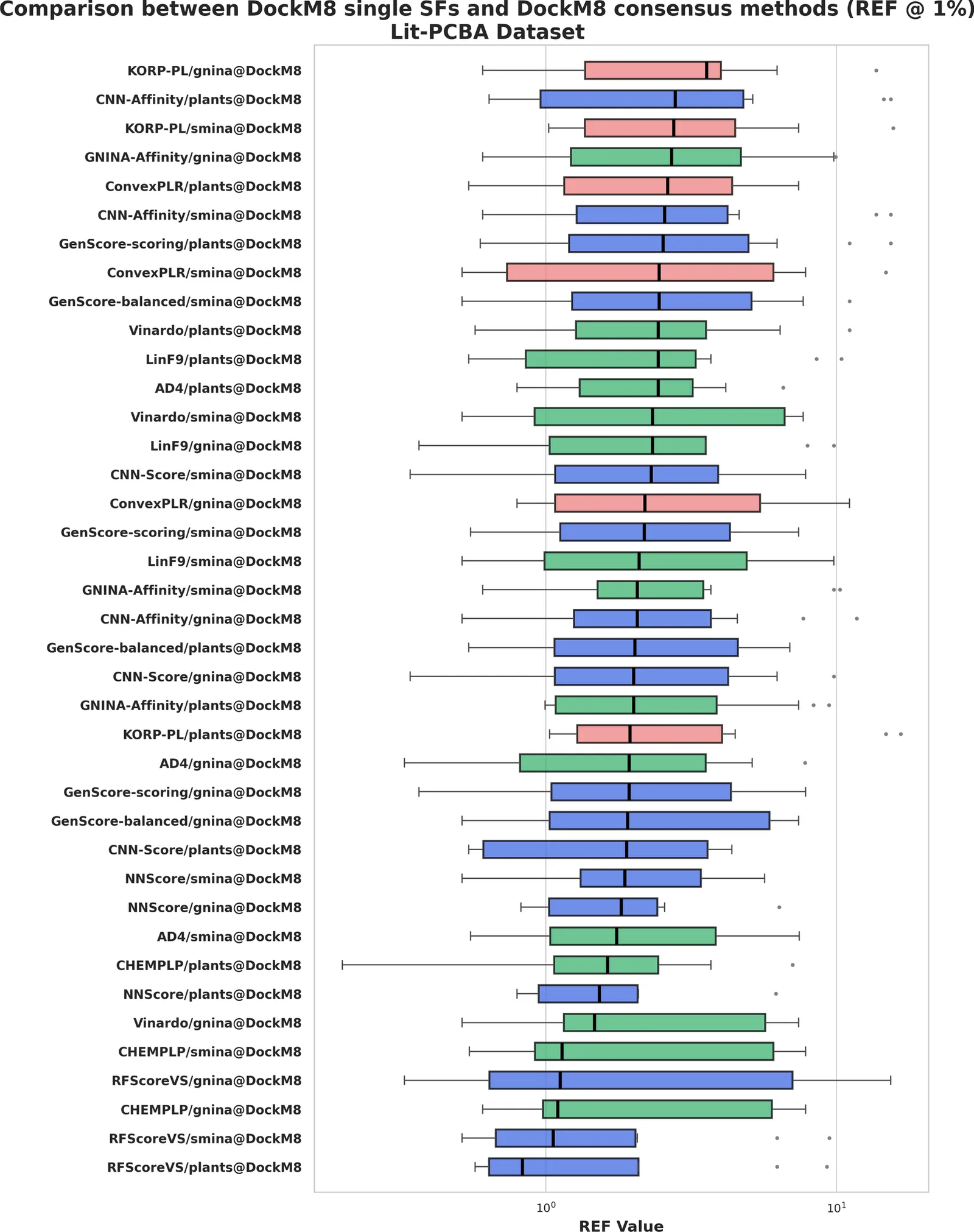
Single scoring functions *vs* consensus methods on Lit-PCBA. Comparison by $\hbox {REF}_{1\%}$. Machine-learning scoring functions are shown in blue, empirical scoring funcions in green, knowledge-based scoring functions in light red, and consensus methods in orange

**Fig. 13:**
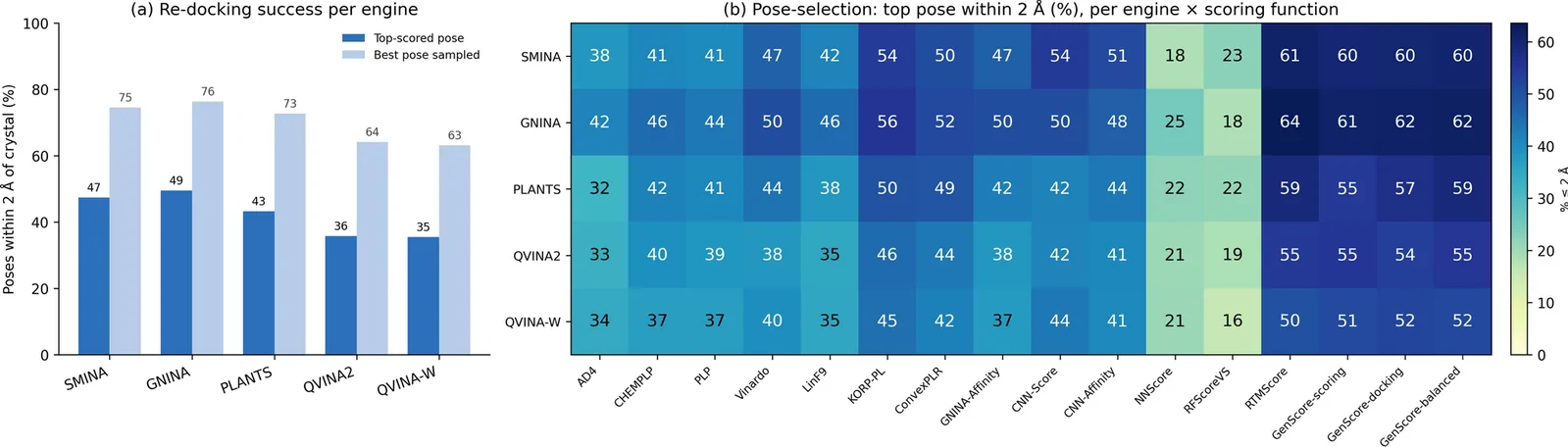
Binding-pose reproduction on the CASF-2016 core set (cognate re-docking). **a** Fraction of poses within 2 Å of the crystal structure for each docking engine: the top-scored pose (dark blue) and the best pose sampled (light blue; sampling ceiling). **b** The same 2 Å success rate for each docking engine paired with each of sixteen SFs used for pose-selection; cell values are percentages.

## Data Availability

All prepared molecules, docking poses, pose-selection results, rescoring and consensus results, and performance metrics are deposited on Zenodo. Owing to the total size of the processed data, the deposits are split across multiple records, one per benchmark dataset (with Lit-PCBA further split into three parts): DEKOIS 2.0: https://doi.org/10.5281/zenodo.15430057. DUD-E: https://doi.org/10.5281/zenodo.15430185. Lit-PCBA (Part 1/3): https://doi.org/10.5281/zenodo.16436210. Lit-PCBA (Part 2/3): https://doi.org/10.5281/zenodo.16436303 Lit-PCBA (Part 3/3): https://doi.org/10.5281/zenodo.16436305. A full description of the contents of each record is provided on the corresponding Zenodo page. Instructions for reproducing the benchmark data from the raw sources are provided in the DockM8 GitHub repository (https://github.com/DrugBud-Suite/DockM8). The raw benchmark datasets themselves are available from their original sources: DEKOIS at https://www.pharmchem.uni-tuebingen.de/dekois/, DUD-E at https://dude.docking.org/, and Lit-PCBA at https://drugdesign.unistra.fr/LIT-PCBA/index.html/.

## Appendix A: Comparison of DockM8 single SFs and consensus methods

See Figs. 10, 11, 12.

## Appendix B: Implementation details

### Software and library versions

For full reproducibility, Table 3 lists the exact versions of the DockM8 platform, the docking engines, the scoring/pose-selection tools, and the principal third-party Python libraries used in this work. The complete environment specification is additionally distributed with the DockM8 source code.

**Table 3:** Exact versions of the software and principal libraries used in this work

Component	Version
*Platform and environment*	
DockM8	v1.1.1
Python	3.10
*Docking engines*	
GNINA	v1.1 (master:e4cb380)
SMINA	via GNINA v1.1 (Vina/Vinardo scoring)
QuickVina-W	1.1
QuickVina 2	2.1
PLANTS	1.2
*Scoring functions/pose selectors*	
RTMScore	GitHub main (retrieved 2024)
GenScore	git commit 5c392bd
KORP-PL	v0.1.2.2
Convex-PLR	v0.5
LinF9	SMINA-based build (2020-11-13; Vina 1.1.2)
SCORCH	1.0.0
RF-Score-VS	ODDT-based (see ODDT)
AA-Score	GitHub main
Gypsum-DL (ligand prep.)	1.2.1
*Principal Python libraries*	
RDKit	2023.09
OpenBabel	3.1.1
ODDT	0.7
Meeko	0.5.1
spyRMSD	0.8.0
scikit-learn	1.2.2
XGBoost	2.1.1
NumPy	1.26.4
pandas	2.1.4
SciPy	1.14.1
TensorFlow	2.18
OpenMM	8.1.2
PDBFixer	1.9
ProDy	2.4.1
Biopython	1.79
PoseBusters	0.3.1
ChEMBL Structure Pipeline	1.2.0
MolVS	0.1.1
BioPandas	0.5.1

SMINA docking is performed using the GNINA executable in its built-in Vina/Vinardo scoring mode. Where a tool is distributed without a formal release tag, the git commit or download source is given instead

### Detailed description and implementation of docking algorithms

DockM8 incorporates the following docking algorithms:

#### SMINA

SMINA is a fork of AutoDock Vina with an improved SF and energy minimization processes [21].

Implementation: SMINA is called directly using its command-line interface. The binding site, defined by the center and size parameters, is passed to SMINA along with the prepared ligand and receptor files.

#### GNINA

GNINA, an offshoot of SMINA, employs convolutional neural networks (CNN) as a SF [3].

Implementation: GNINA is executed via its command-line interface. The CNN SF is enabled by default, and the binding site parameters are passed similarly to SMINA.

#### QVINA-W and QVINA2

Both derived from AutoDock Vina [6], QVINA-W [61] and QVINA2 [62] introduce advancements in computational speed, expediting the docking process.

Implementation: QVINA-W and QVINA2 require input files in PDBQT format. DockM8 uses Meeko to convert ligands and MGLTools (prepare_receptor4.py from AutoDockTools, executed in a dedicated conda environment) to convert receptors to PDBQT format before docking. The docking is then performed using the respective command-line interfaces.

#### PLANTS

PLANTS is based on an ant colony optimization algorithm [63].

Implementation: PLANTS is executed through its command-line interface. DockM8 generates a configuration file for each docking run, specifying the binding site, SF, and other parameters.

### Detailed description and implementation of pose-selection methods

Any of the 17 SFs supported by DockM8 can be used to select docking poses. In this case, the best scoring pose for each ligand is used for further calculations.

Implementation: DockM8 applies the selected SF to all poses of a ligand and selects the pose with the best score. This process is repeated for each ligand in the dataset.

## Appendix C: Virtual screening performance metrics

The following mathematical formulations are used in the performance evaluation metrics implemented in the DockM8 workflow:

### Notation

- *N*: Total number of compounds
- $N_s$: Number of compounds in selection (at a given percentile threshold)
- *n*: Total number of active compounds
- $n_s$: Number of active compounds in selection

### Threshold-dependent metrics

#### Enrichment factor (EF)

7 $$\begin{aligned} EF = \frac{n_s / N_s}{n / N} = \frac{N \cdot n_s}{n \cdot N_s} \end{aligned}$$

#### Relative enrichment factor (REF)

8 $$\begin{aligned} REF = 100 \cdot \frac{n_s}{\min (N_s, n)} \end{aligned}$$

#### Power Metric (PM)

9 $$\begin{aligned} PM = \frac{n_s \cdot N - n \cdot n_s}{n_s \cdot N - 2 \cdot n \cdot n_s + n \cdot N_s} \end{aligned}$$

#### ROC Enrichment (ROCE)

10 $$\begin{aligned} ROCE = \frac{TPR}{FPR} = \frac{n_s/n}{(N_s-n_s)/(N-n)} = \frac{n_s \cdot (N-n)}{n \cdot (N_s-n_s)} \end{aligned}$$

#### Correct Classification Rate (CCR)

11 $$\begin{aligned} & CCR = \frac{Sensitivity + Specificity}{2} = \frac{1}{2} \left( \frac{n_s}{n}\right. \nonumber \\ & \left. + \frac{(N-N_s-n+n_s)}{(N-n)} \right) \end{aligned}$$

#### Matthews correlation coefficient (MCC)

12 $$\begin{aligned} MCC = \frac{N \cdot n_s - N_s \cdot n}{\sqrt{N_s \cdot n \cdot (N-n) \cdot (N-N_s)}} \end{aligned}$$

#### Cohen’s kappa coefficient (CKC)

13 $$\begin{aligned} CKC = 1 - \frac{N \cdot n + N \cdot N_s - 2 \cdot n_s \cdot N}{N \cdot n + N \cdot N_s - 2 \cdot n \cdot N_s} \end{aligned}$$

### Threshold-independent metrics

#### Area under the ROC curve (AUC-ROC)

AUC-ROC is calculated by integrating the area under the Receiver Operating Characteristic curve, which plots the true positive rate against the false positive rate at various threshold settings. This is implemented using the roc_auc_score function from the scikit-learn library.

#### Area under the precision-recall curve (AUPR)

AUPR is calculated by integrating the area under the Precision-Recall curve, which plots precision against recall at various threshold settings. This is implemented using the average_precision_score function from the scikit-learn library.

#### Boltzmann-enhanced discrimination of ROC (BEDROC)

14 $$\begin{aligned} BEDROC = \frac{RIE - RIE_{min}}{RIE_{max} - RIE_{min}} \end{aligned}$$

Where RIE is the Robust Initial Enhancement (see below) and $RIE_{min}$ and $RIE_{max}$ are the minimum and maximum possible values of RIE. This is implemented using the CalcBEDROC function from the RDKit library.

#### Robust initial enhancement (RIE)

15 $$\begin{aligned} RIE = \frac{1}{n} \sum _{i=1}^{n} e^{-\alpha r_i / N} \end{aligned}$$

Where $r_i$ is the rank of the *i*-th active compound, and α is a parameter that controls the emphasis on early recognition. We set α=80.5 throughout; this concentrates the early-recognition weight on the top ${\sim }2\%$ of the ranked list, making BEDROC approximately analogous to an enrichment factor evaluated at 2% ($\hbox {EF}_{2\%}$), following the analysis of Truchon and Bayly [77]. This is implemented using the CalcRIE function from the RDKit library.

### Score normalization

Before metrics calculation, all SFs are normalized to a common scale (0-1), where 1 always represents the best score:

16 $$\begin{aligned} normalized\_score = \frac{score - min\_score}{max\_score - min\_score} \end{aligned}$$

For SFs where lower values are better:

17 $$\begin{aligned} normalized\_score = 1 - \frac{score - min\_score}{max\_score - min\_score} \end{aligned}$$

## Appendix D: Subset of targets selected for each dataset

See Tables 4, 5, 6.

**Table 4:** Targets selected from DEKOIS 2.0 dataset (79 targets)

11BETAHSD1	FKBP1A	PDK1
17BETAHSD1	FXA	PI3KG
A2A	GBA	PIM-1
ACE	GR	PIM-2
ACE2	GSK3B	PNP
ACHE	HDAC2	PPARA
ADAM17	HDAC8	PPARG
ADRB2	HIV1PR	PR
AKT1	HIV1RT	PYGL-IN
ALR2	HMGR	PYGL-OUT
AR	HSP90	QPCT
AURKA	INHA	ROCK-1
AURKB	ITK	RXR
BCL2	JAK3	SARS-HCOV
BRAF	JNK1	SIRT2
CATL	JNK2	SRC
CDK2	JNK3	THROMBIN
COX1	KIF11	TIE2
COX2	LCK	TK
CTSK	MDM2	TP
CYP2A6	MK2	TPA
DHFR	MMP2	TS
EGFR	NA	UPA
EPHB4	P38-ALPHA	VEGFR1
ER-BETA	PARP-1	VEGFR2
ERBB2	PDE4B	
FGFR1	PDE5	

All targets contain 40 actives and 1200 decoys

**Table 5:** Targets selected from DUD-E dataset (28 targets) with corresponding number of actives and decoys

Target	Actives	Decoys	Target	Actives	Decoys	Target	Actives	Decoys
ABL1	182	10,746	FABP4	47	2,749	ITAL	138	8,487
ACE	282	16,860	GRIA2	158	11,832	KITH	57	2,850
ALDR	159	8,995	HDAC2	185	10,299	KPCB	135	8,692
ANDR	269	14,343	HIVRT	338	18,879	LKHA4	171	9,448
CDK2	474	27,830	HMDH	170	8,743	NRAM	98	6,199
DHI1	330	19,340	HS90A	88	4,848	PGH1	195	10,797
DYR	231	17,157	HXK4	92	4,696	PRGR	293	15,642
ESR1	383	20,663	IGF1R	148	9,291	PTN1	130	7,243
FA7	114	6,245				PYRD	111	6,446
						RENI	104	6,955
						UROK	162	9,841

**Table 6:** Targets selected from Lit-PCBA dataset (14 targets) with corresponding number of actives and inactives

Target	Actives	Inactives	Target	Actives	Inactives
ADRB2	17	311,748	KAT2A	194	342,729
ALDH1	5,363	101,874	MAPK1	308	61,567
ESR1A	13	4,378	MTORC1	97	32,972
ESR1T	88	3,820	PKM2	546	244,679
FEN1	360	350,718	PPARG	24	4,071
GBA	163	291,241	TP53	64	3,345
IDH1	39	358,757	VDR	655	262,648

## Appendix E: AVE set compound counts for Lit-PCBA targets

See Table 7.

**Table 7:** AVE set compound counts for Lit-PCBA targets, split into training and validation sets as provided by the original Lit-PCBA authors

Target	Training set			Validation set		
	Active	Inactive	Total	Active	Inactive	Total
ADRB2	13	233,957	233,970	4	77,791	77,795
ALDH1	4,020	76,577	80,597	1,343	25,297	26,640
ESR1A	10	3,470	3,480	3	908	911
ESR1T	63	3,026	3,089	25	794	819
FEN1	269	263,771	264,040	91	86,947	87,038
GBA	122	219,042	219,164	41	72,199	72,240
IDH1	30	269,664	269,694	9	89,093	89,102
KAT2A	146	258,067	258,213	48	84,662	84,710
MAPK1	231	46,317	46,548	77	15,250	15,327
MTORC1	73	24,729	24,802	24	8,243	8,267
PKM2	410	183,672	184,082	136	61,007	61,143
PPARG	18	3,210	3,228	6	861	867
TP53	48	2,550	2,598	16	795	811
VDR	490	197,557	198,047	165	65,091	65,256

## Appendix F: DockM8 conditions for the DEKOIS 2.0, DUD-E and Lit-PCBA benchmark datasets

### DEKOIS 2.0

The DockM8 conditions were set as follows:

- Protein preparation: the protein file was protonated using the –prepare_protein True option.
- Pocket definition: the pocket was defined using the –pocket_method Reference option with –pocket_radius 8 (yielding a 16 Å cubic box).
- Ligand preparation: the ligands were protonated and 3D conformers were generated using Gypsum-DL.
- Docking: PLANTS, SMINA, GNINA, QVINA2, QVINAW: in all cases, 10 poses per ligand were generated.
- Pose-selection: due to the high computational cost of exploring each SF as a pose-selection tool, we elected to use only the following methods: bestpose_GNINA, bestpose_SMINA, bestpose_PLANTS, bestpose_QVINA2, bestpose_QVINAW (the top-scoring pose from each docking engine), and the following SFs used as pose selectors: AD4, CHEMPLP, CNN-Affinity, CNN-Score, ConvexPLR, GNINA-Affinity, GenScore-balanced, GenScore-docking, GenScore-scoring, KORP-PL, LinF9, NNScore, RFScoreVS, RTMScore, Vinardo.
- Pose busting: Due to the high computational cost of using Pose Busters on this many docking poses, this feature was disabled.
- Rescoring: GNINA-Affinity, CNN-Score, CNN-Affinity, Vinardo, AD4, KORP-PL, ConvexPLR, LinF9, RFScoreVS, CHEMPLP, NNScore, GenScore-balanced, GenScore-docking, GenScore-scoring, RTMScore (SCORCH was omitted in the benchmarking used due to high computational cost. PLP was omitted due to high correlation with CHEMPLP).
- Consensus: for benchmarking purposes, every consensus method available in DockM8 was used (ecr, rbr, rbv, soft_rbv, zscore). Moreover, the consensus was considered for every pose-selection method and every combination of the selected SFs. This represents 16 376 000 possible final compound rankings for each target in the dataset (excludes empty SF sets and singletons, consistent with section Asymmetric validation embedding analysis).
- Resulting DockM8-median configuration: the single workflow achieving the highest median $\hbox {REF}_{1\%}$ across DEKOIS 2.0 targets used the GNINA docking engine, GenScore-balanced pose-selection, zscore consensus, and the scoring-function set {AD4, CHEMPLP, CNN-Score, GenScore-balanced, GenScore-docking, KORP-PL, RFScoreVS, RTMScore}.

### DUD-E

The DockM8 conditions were set as follows:

- Protein preparation: the protein file was protonated using the –prepare_protein True option.
- Pocket definition: the pocket was defined using the –pocket_method Reference option with –pocket_radius 8 (yielding a 16 Å cubic box).
- Ligand preparation: the ligands were protonated and 3D conformers were generated using Gypsum-DL.
- Docking: PLANTS, SMINA, GNINA: in all cases, 10 poses per ligand were generated.
- Pose-selection: due to the high computational cost of exploring each SF as a pose-selection tool, we elected to use only the following methods: bestpose_GNINA, bestpose_SMINA, bestpose_PLANTS (the top-scoring pose from each docking engine), and the following SFs used as pose selectors: AD4, CHEMPLP, CNN-Affinity, CNN-Score, ConvexPLR, GNINA-Affinity, GenScore-balanced, GenScore-docking, GenScore-scoring, KORP-PL, LinF9, NNScore, RFScoreVS, RTMScore, Vinardo.
- Pose busting: Due to the high computational cost of using Pose Busters on this many docking poses, we elected to not enable this feature.
- Rescoring: GNINA-Affinity, CNN-Score, CNN-Affinity, Vinardo, AD4, KORP-PL, ConvexPLR, LinF9, RTMScore, RFScoreVS, CHEMPLP, NNScore, GenScore-balanced, GenScore-docking, GenScore-scoring (SCORCH was omitted in the benchmarking used due to high computational cost. PLP was omitted due to high correlation with CHEMPLP).
- Consensus: for benchmarking purposes, every consensus method available in DockM8 was used (ecr, rbr, rbv, soft_rbv, zscore). Moreover, the consensus was considered for every pose-selection method and every combination of the selected SFs. This represents 8 843 040 possible final compound rankings for each target in the dataset (excludes empty SF sets and singletons, consistent with Section Asymmetric validation embedding analysis).
- Resulting DockM8-median configuration: the single workflow achieving the highest median $\hbox {REF}_{1\%}$ across DUD-E targets used the PLANTS docking engine, GenScore-scoring pose-selection, zscore consensus, and the scoring-function set {CNN-Score, GenScore-balanced, GenScore-scoring, KORP-PL, RFScoreVS, Vinardo}.

### Lit-PCBA

The DockM8 conditions were set as follows:

- Protein preparation: the protein file was protonated using the –prepare_protein True option.
- Pocket definition: the pocket was defined using the –pocket_method Reference option with –pocket_radius 8 (yielding a 16 Å cubic box).
- Ligand preparation: the ligands were protonated and 3D conformers were generated using Gypsum-DL.
- Docking: PLANTS, SMINA, GNINA: in all cases, 10 poses per ligand were generated.
- Pose-selection: due to the high computational cost of exploring all the pose-selection methods, we elected to use only the following methods: bestpose_GNINA, bestpose_SMINA, bestpose_PLANTS (the top-scoring pose from each docking engine), and the following SFs used as pose selectors: AD4, CHEMPLP, CNN-Affinity, CNN-Score, ConvexPLR, GNINA-Affinity, GenScore-balanced, GenScore-docking, GenScore-scoring, KORP-PL, LinF9, NNScore, RFScoreVS, RTMScore, Vinardo.
- Pose busting: Due to the high computational cost of using Pose Busters on this many docking poses, we elected to not enable this feature.
- Rescoring: GNINA-Affinity, CNN-Score, CNN-Affinity, Vinardo, AD4, KORP-PL, ConvexPLR, LinF9, RFScoreVS, CHEMPLP, NNScore, GenScore-balanced, GenScore-docking, GenScore-scoring, RTMScore (SCORCH was omitted in the benchmarking used due to high computational cost. PLP was omitted due to high correlation with CHEMPLP).
- Consensus: for benchmarking purposes, every consensus method available in DockM8 was used (ecr, rbr, rbv, soft_rbv, zscore). Moreover, the consensus was considered for every pose-selection method and every combination of the selected SFs. This represents 8 843 040 possible final compound rankings for each target in the dataset (excludes empty SF sets and singletons, consistent with Section Asymmetric validation embedding analysis).
- Resulting DockM8-median configuration: the single workflow achieving the highest median $\hbox {REF}_{1\%}$ across Lit-PCBA targets used the PLANTS docking engine, CNN-Score pose-selection, rbv consensus, and the scoring-function set {AD4, ConvexPLR, GNINA-Affinity, GenScore-balanced, GenScore-docking, GenScore-scoring, KORP-PL, RTMScore, Vinardo}.

## Appendix G: Binding-pose reproduction on the CASF-2016 core set

While the main text evaluates DockM8 by screening (enrichment) power, the ability of a docking protocol to reproduce the experimentally observed binding pose is a complementary and equally important measure of SBVS quality. To characterize this for DockM8, we performed a cognate re-docking experiment on the CASF-2016 core set, which comprises 285 high-quality protein–ligand crystal structures spanning diverse targets [44]. We state the scope of this experiment plainly: it is a cognate (self-)re-docking benchmark, in which each ligand is re-docked into its own co-crystal receptor with the search box centered on the crystallographic ligand. The reported rates therefore quantify the engines’ and pose selectors’ ability to recover a known binding mode under favorable conditions and represent an upper bound relative to cross-docking or fully prospective (blind) docking. They are also not a direct measure of pose accuracy on the screening actives of section Assessment of screening power on DEKOIS 2.0, DUD-E and Lit-PCBA, which lack experimentally determined bound poses; cognate re-docking on crystallographic complexes is the standard available proxy.

### Protocol

For each complex, the receptor was prepared with the standard DockM8 protein-preparation pipeline (including Protoss protonation) and the binding site was defined from the co-crystallised ligand (10 Å radius). To avoid biasing the result with the crystallographic coordinates, the ligand was treated as in a prospective run: its 3D conformer was regenerated from scratch with DockM8’s library-preparation step (Gypsum-DL [53], fixed random seed) and then re-docked. Each of the five docking engines (SMINA, GNINA, PLANTS, QVINA2, QVINA-W) generated up to ten poses per ligand (exhaustiveness 8). For every engine, the top pose was selected both by the engine’s native score (the “top-scored” pose) and, independently, by each of sixteen SFs used as pose selectors (all seventeen DockM8 SFs except SCORCH, which was omitted for computational cost). The heavy-atom RMSD of each selected pose to the crystal ligand was computed with a symmetry-corrected, superposition-free metric (spyRMSD [78]); because the docked pose and the crystal ligand share the receptor frame, no rigid-body alignment is applied. A pose is counted as a success if its RMSD is ≤2 Å, the standard threshold used in pose-prediction benchmarks [44, 79].

### Results

Figure 13a reports, for each engine, the fraction of top-scored poses within 2 Å of the crystal structure, alongside the fraction for which *any* sampled pose is within 2 Å (the sampling ceiling). GNINA and SMINA reproduced the crystallographic pose most often (top-scored success of 49.5% and 47.3%, respectively), followed by PLANTS (43.3%), with the QVINA variants lower (≈36%). The substantial gap between the top-scored bars and the sampling ceiling (63–76%) shows that all engines frequently sample a near-native pose that their own score does not rank first, motivating the use of dedicated pose-selection SFs. Figure 13b breaks the 2 Å success rate down by engine and pose-selection SF: the deep-learning RTMScore and GenScore were the strongest pose selectors across engines (up to ${\approx }\,64\%$), followed by the knowledge-based KORP-PL and ConvexPLR (up to ${\approx }\,56\%$ and ${\approx }\,52\%$, respectively); all four recovered more near-native top poses than the engines’ native scores. Table 8 summarizes the per-engine figures. Overall, DockM8’s docking engines reproduce native binding poses at rates consistent with established docking tools on this benchmark [44], and its pose-selection layer can further improve near-native pose recovery.

**Table 8:** Per-engine binding-pose reproduction on the CASF-2016 core set. “Top-scored” is the success rate of the pose ranked first by the engine’s native score; “Best sampled” considers the best of the sampled poses (sampling ceiling); the median RMSD is taken over the top-scored poses. *N* is the number of evaluable complexes.

Engine	*N*	Top-scored ≤2 Å (%)	Best sampled ≤2 Å (%)	Median RMSD (Å)
GNINA	283	49.5	76.3	2.05
SMINA	283	47.3	74.6	2.22
PLANTS	275	43.3	72.7	2.98
QVINA2	279	35.8	64.2	3.11
QVINA-W	279	35.5	63.1	3.21

### Practical considerations

A few practical points are worth noting. (i) This is a cognate (self-) re-docking experiment with the search box centered on the crystal ligand; the reported rates are therefore an upper bound relative to cross- or blind docking. (ii) Conversion of the prepared receptor to the AutoDock PDBQT format (required by the QVINA engines) failed for a subset of receptors with the default tool; these cases were handled transparently with an OpenBabel [80] fallback. (iii) A small number of complexes could not be evaluated for individual engines and were excluded rather than counted as failures: docked poses of some amidine/guanidinium-containing ligands could not be re-parsed for PLANTS (10/285), and a few macrocyclic ligands were incompatible with the QVINA ligand atom typing (6/285); the per-engine *N* is given in Table 8.
