# Supplementary material for: DockM8: an all-in-one open-source platform for consensus virtual screening in drug design
Source: J Cheminform. 2026 Sep 18;18:135. doi: 10.1186/s13321-026-01287-2 (PMC13613745; doi:10.1186/s13321-026-01287-2)
Supplement: Supplementary file 1 [file 13321_2026_1287_MOESM2_ESM.pdf]

Supporting Information for DockM8: An All-in-One Open-Source Platform for Consensus Virtual Screening in Drug Design

Antoine Lacour[1,2,3], Hamza Agha[3], Anna K. H. Hirsch[1,2*], Andrea Volkamer[1,3*]

[1]Helmholtz Institute for Pharmaceutical Research Saarland (HIPS), Helmholtz Centre for Infection Research (HZI), Saarbrücken, Germany.
[2]Department of Pharmacy, Saarland University, Saarbrücken, Germany.
[3]Data Driven Drug Design, Faculty of Mathematics and Computer Sciences, Saarland University, Saarbrücken, Germany.

*Corresponding author(s). E-mail(s): anna.hirsch@helmholtz-hips.de; andrea.volkamer@uni-saarland.de;

Abstract

Keywords: Virtual screening, Consensus docking, Molecular docking, Drug design, Open source

Contents

1 Comparison to Literature Methods **2**
1.1 AUC-ROC 2
1.2 BEDROC 3
1.3 Enrichment Factor at 1% 4
1.4 Power Metric at 1% 5
1.5 Relative Enrichment Factor at 1% 7

2 Generalizability Assessment **9**

1 Comparison to Literature Methods

The following figures compare DockM8 performance against published protocols across the Demanding Evaluation Kits for Objective In Silico Screening (DEKOIS), Directory of Useful Decoys - Enhanced (DUD-E), and Lit-PCBA datasets. In all figures, methods are sorted by their median value and whiskers denote the 10th and 90th percentiles. The following notation is used throughout: methods containing the @ symbol indicate the provenance of the data (e.g., @IGN, @DockM8); methods containing the / symbol indicate the software used to obtain the docking poses for scoring (e.g., /GlideSP); methods using a consensus approach are highlighted with $*$; methods tested on only a subset of targets are highlighted with † and the number of targets tested is shown in brackets; methods that used the same dataset for training are highlighted with ‡. Within each panel, `DockM8-best` and `DockM8-median` (including RF-Score-VS) are shown in orange, their RF-Score-VS-excluded variants (`no RFScoreVS`) in purple, and the individual scoring functions evaluated within the DockM8 framework (the @DockM8 methods) in grey; the remaining entries are published literature protocols. The RF-Score-VS-excluded variants are shown for DEKOIS 2.0 and DUD-E only, as no corresponding variant was computed for Lit-PCBA.

Four filtering variants are presented for Enrichment Factor (EF), Power Metric (PM), and Relative Enrichment Factor (REF) metrics: (1) standard target set with curated model selection, (2) all published models included, (3) all targets in the dataset included, and (4) all models and all targets combined. This comprehensive evaluation ensures transparency regarding how model and target selection affect comparative performance.

1.1 AUC-ROC

Area under the Receiver Operating Characteristic Curve (AUC-ROC) results across all three datasets are shown in Figure S1.

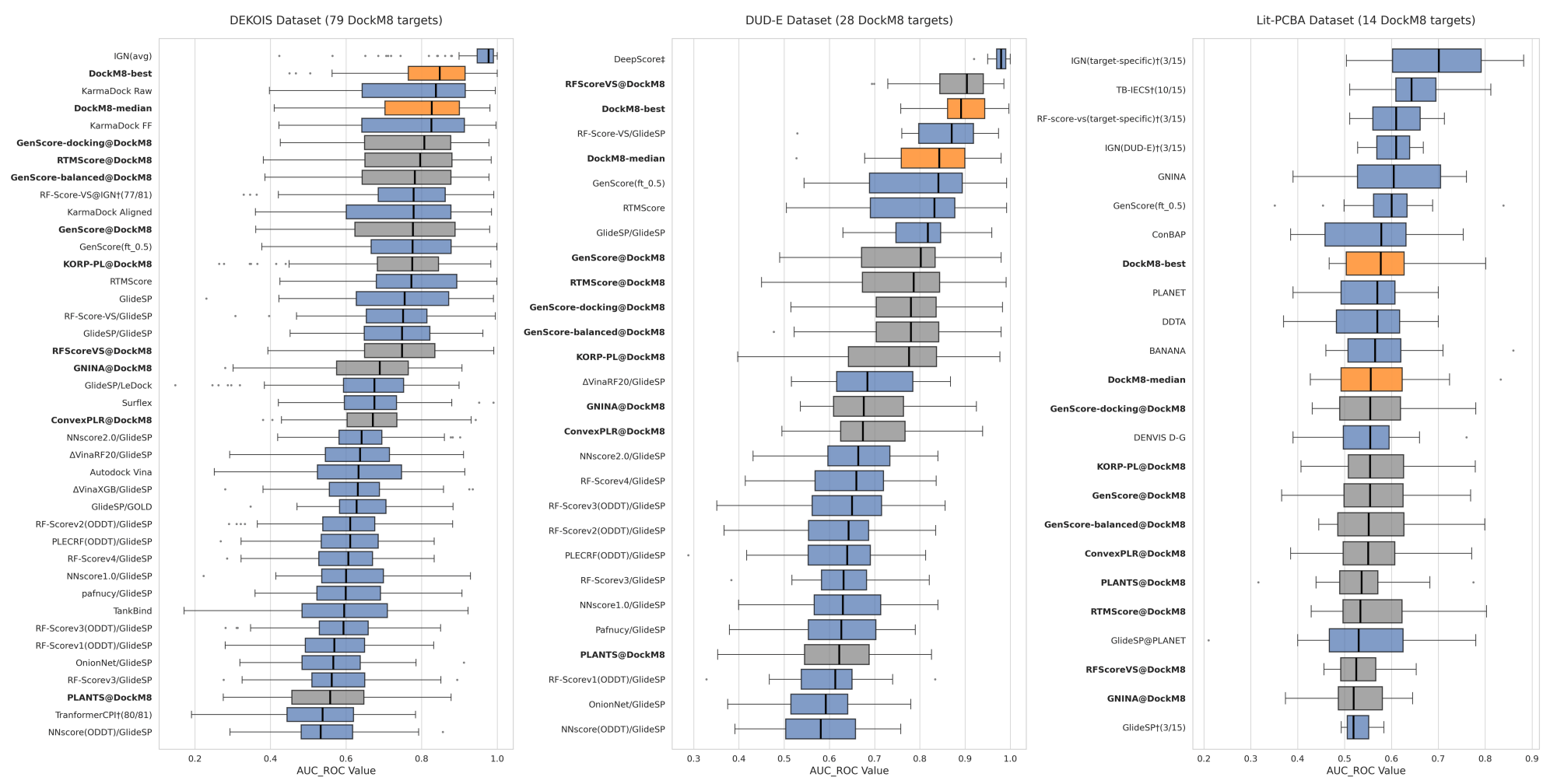

Fig. S1 Evaluation of AUC-ROC of DockM8 compared to published protocols on the DEKOIS, DUD-E, and Lit-PCBA datasets. See Section 1 for notation and methodology.

1.2 BEDROC

Boltzmann-Enhanced Discrimination of ROC (BEDROC) results across all three datasets are shown in Figure S2.

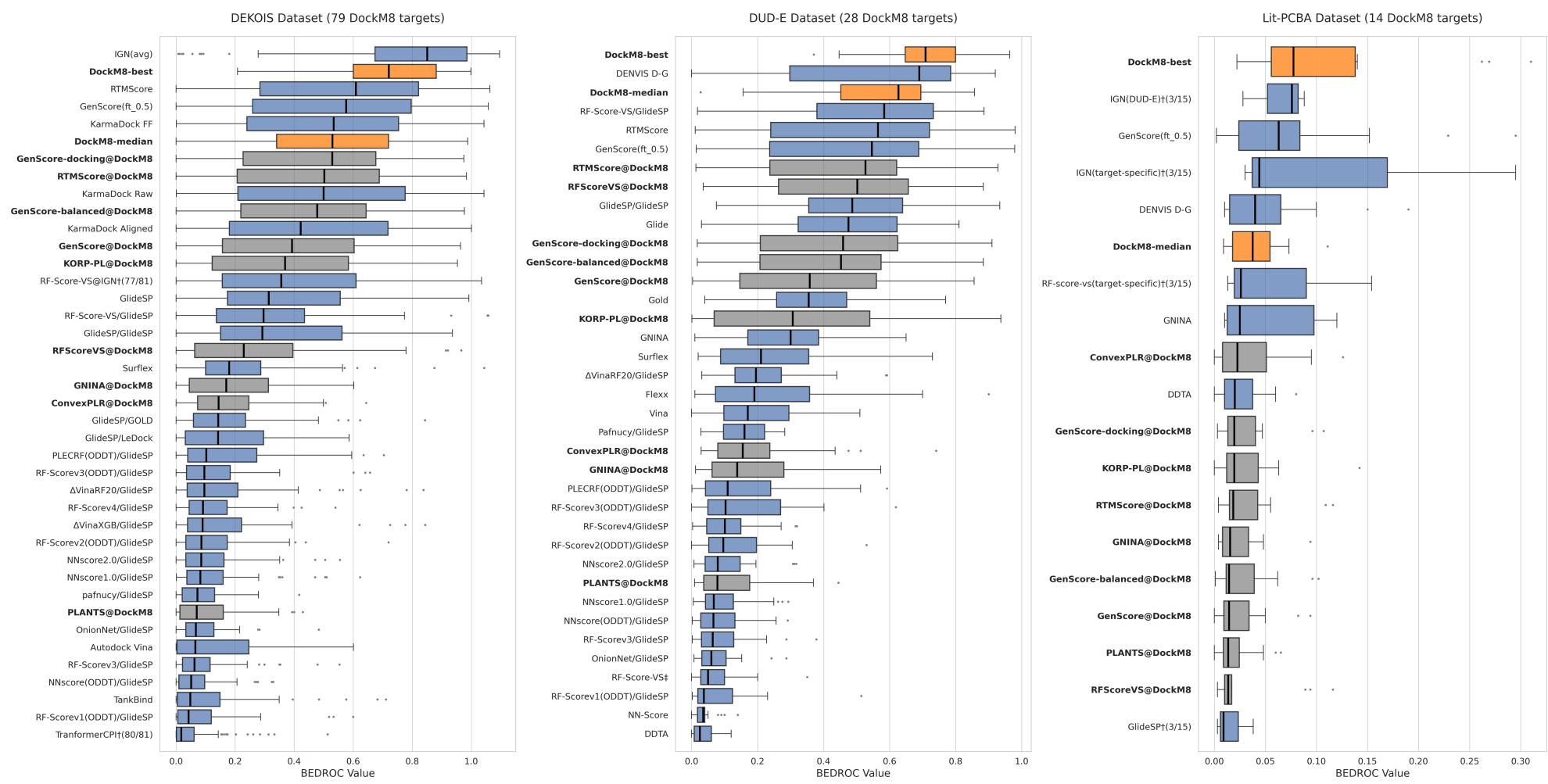

Fig. S2 Evaluation of BEDROC of DockM8 compared to published protocols on the DEKOIS, DUD-E, and Lit-PCBA datasets. BEDROC emphasizes early recognition performance, providing a stronger weighting to compounds ranked at the top of the list. See Section 1 for notation.

1.3 Enrichment Factor at 1%

EF at 1% results are shown across the four variants (Figures S3, S4, S5, and S6).

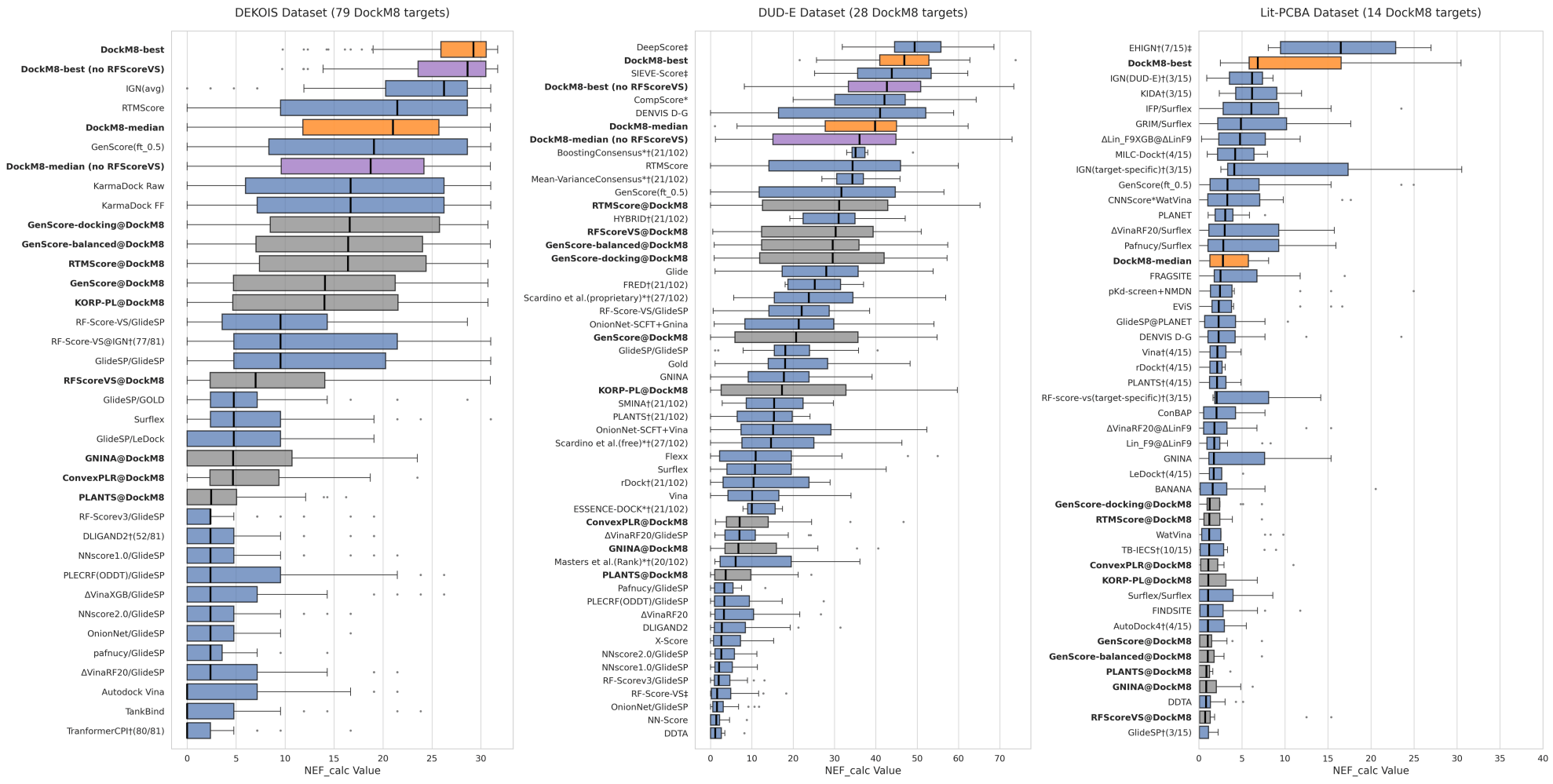

Fig. S3 Evaluation of EF at 1% of DockM8 compared to published protocols on the DEKOIS, DUD-E, and Lit-PCBA datasets using the standard target set. See Section 1 for notation.

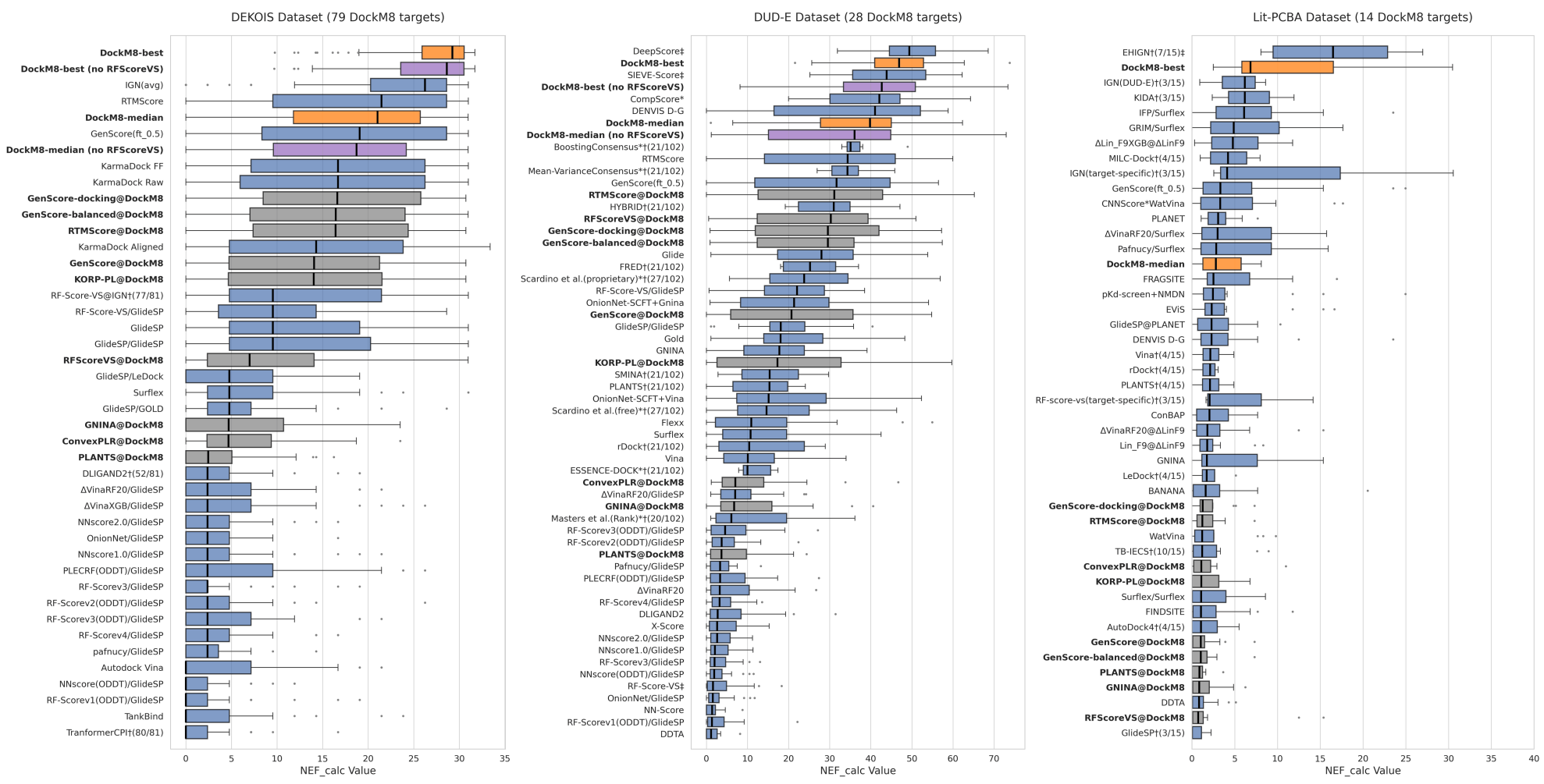

Fig. S4 Evaluation of EF at 1% including all published models for which literature data was obtained, using the standard target set. See Section 1 for notation.

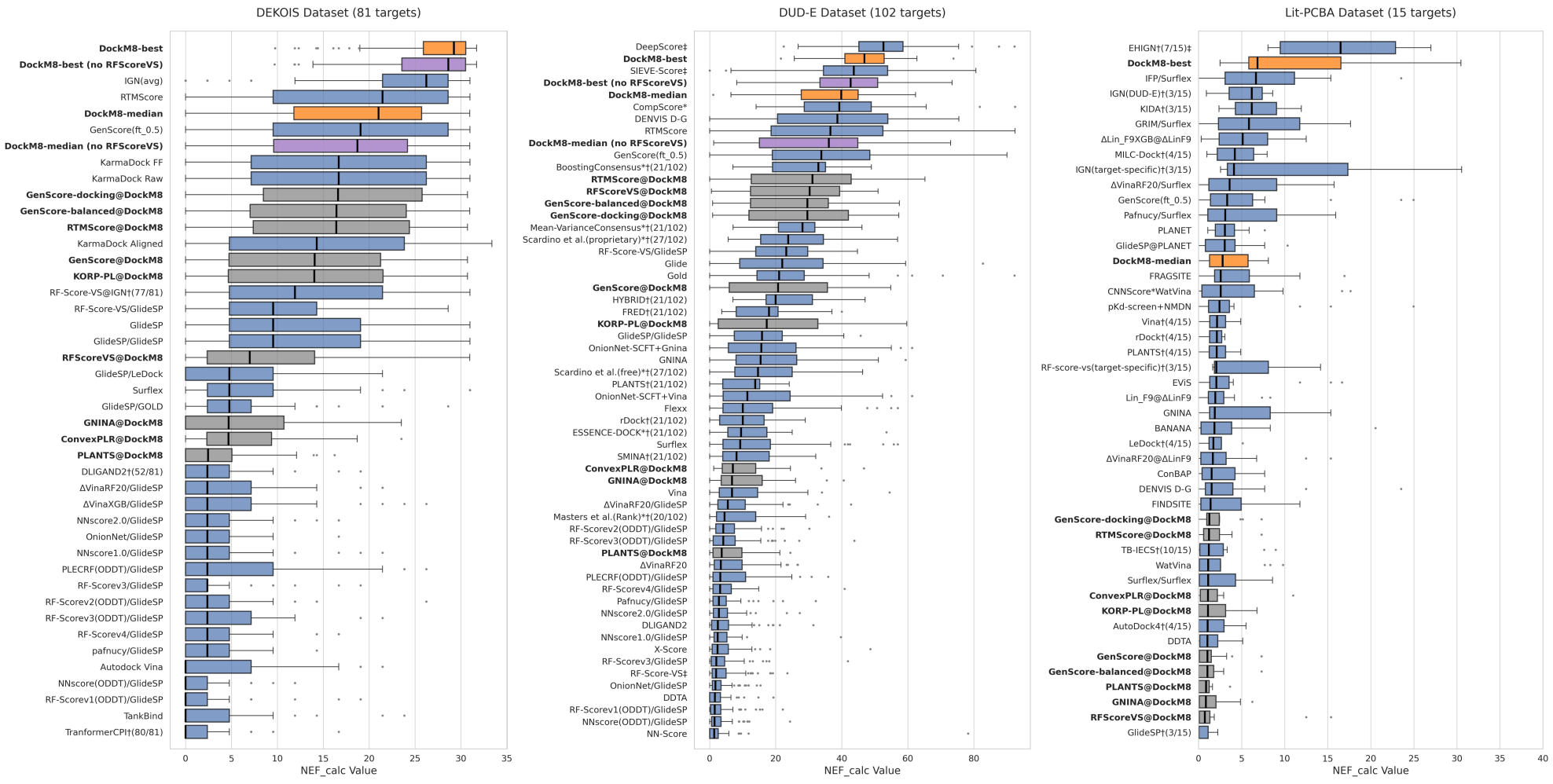

Fig. S5 Evaluation of EF at 1% including all published models and all targets in the datasets. See Section 1 for notation.

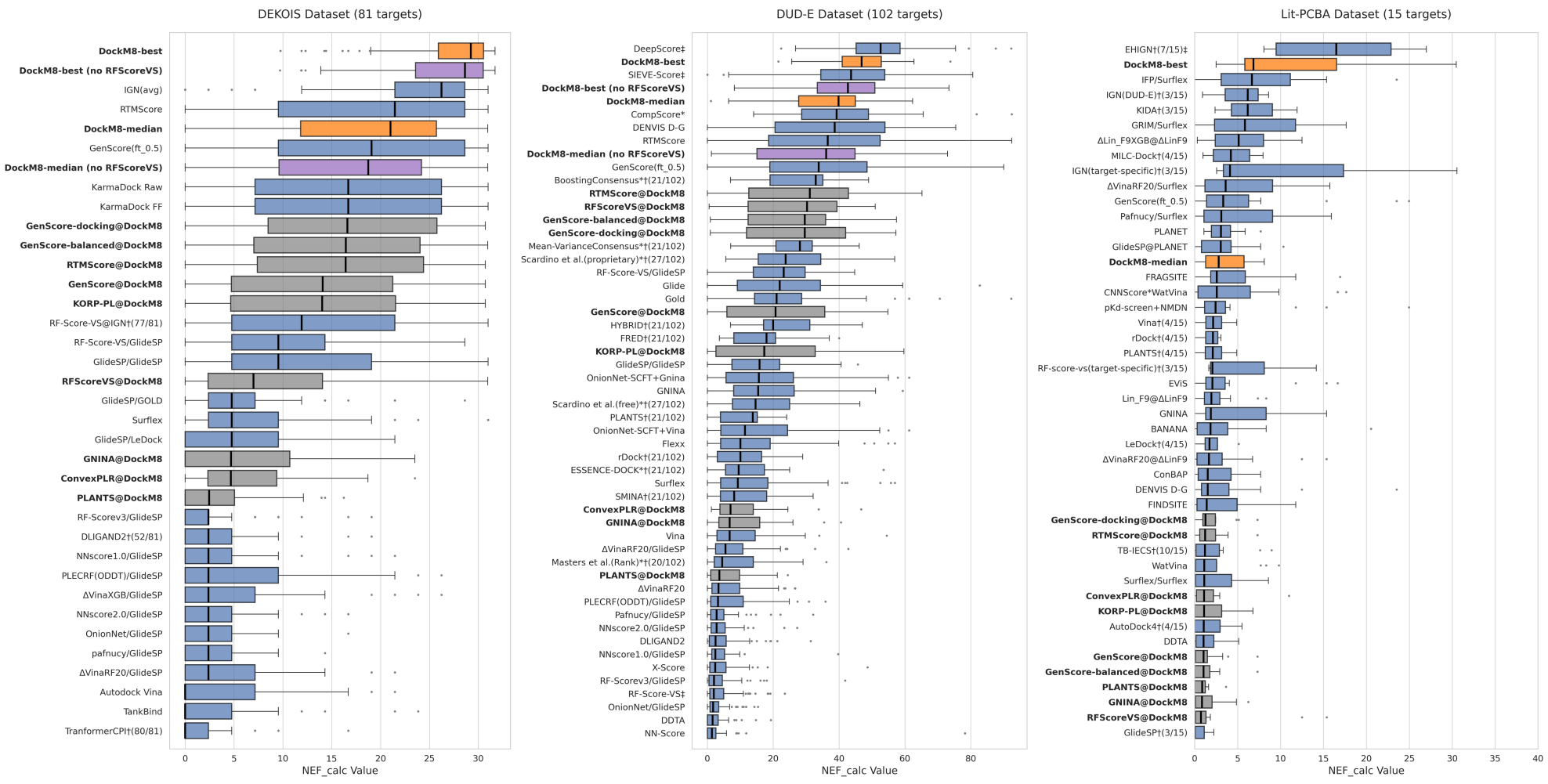

Fig. S6 Evaluation of EF at 1% including all targets in the datasets, not limited to those tested with DockM8. See Section 1 for notation.

1.4 Power Metric at 1%

PM at 1% results are shown across the four variants (Figures S7, S8, S9, and S10).

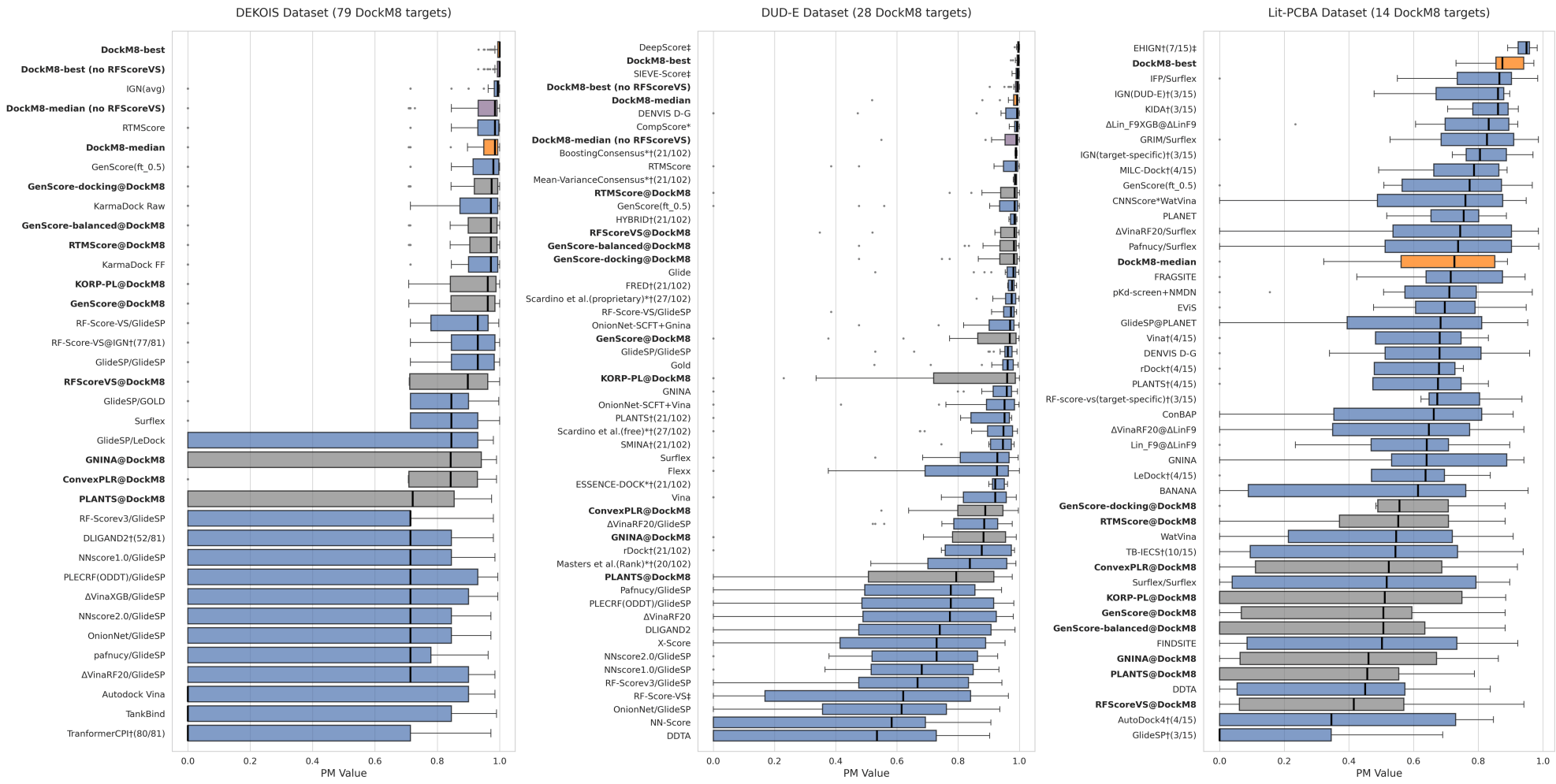

Fig. S7 Evaluation of PM at 1% of DockM8 compared to published protocols on the DEKOIS, DUD-E, and Lit-PCBA datasets using the standard target set. See Section 1 for notation.

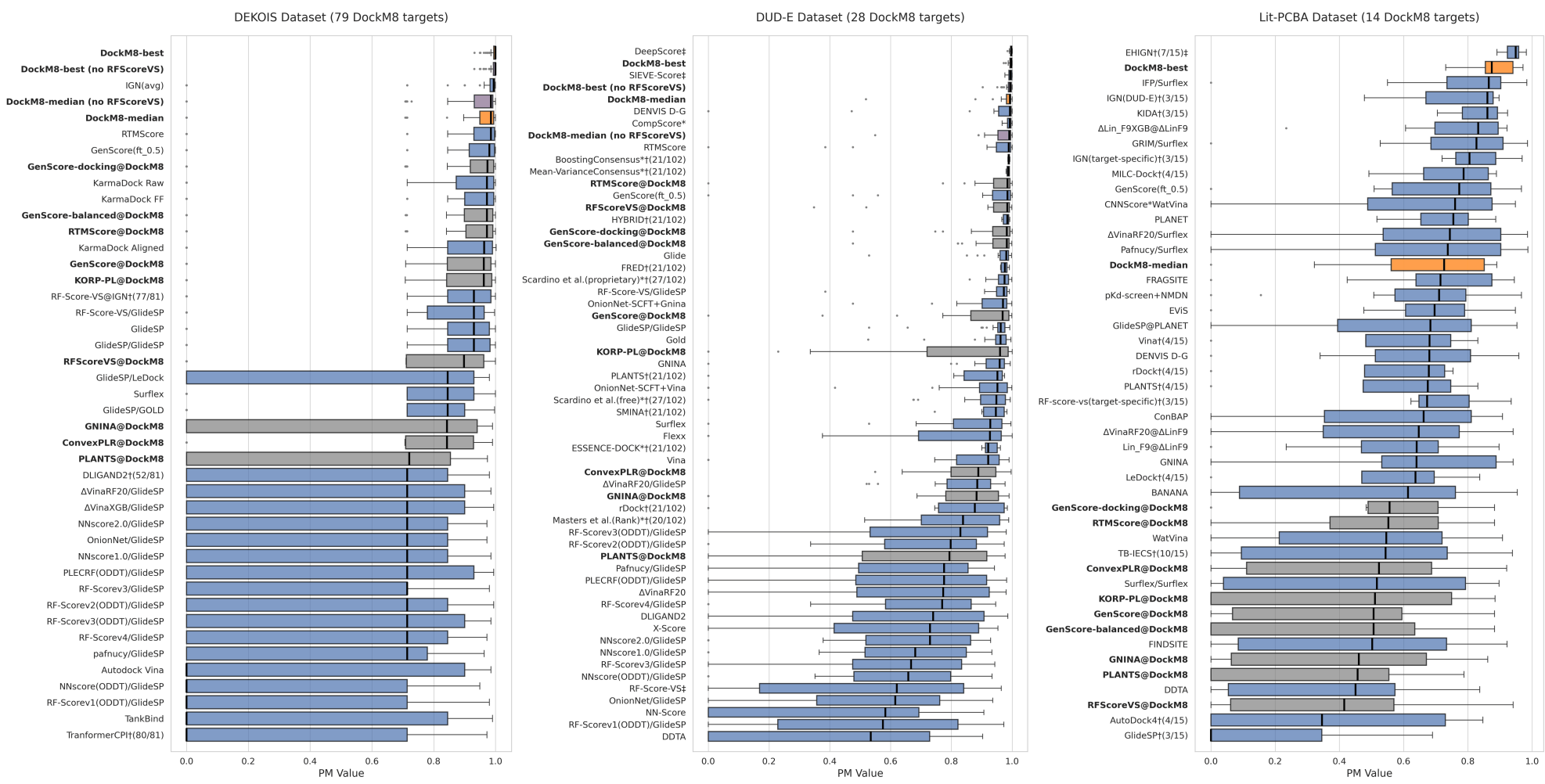

Fig. S8 Evaluation of PM at 1% including all published models for which literature data was obtained, using the standard target set. See Section 1 for notation.

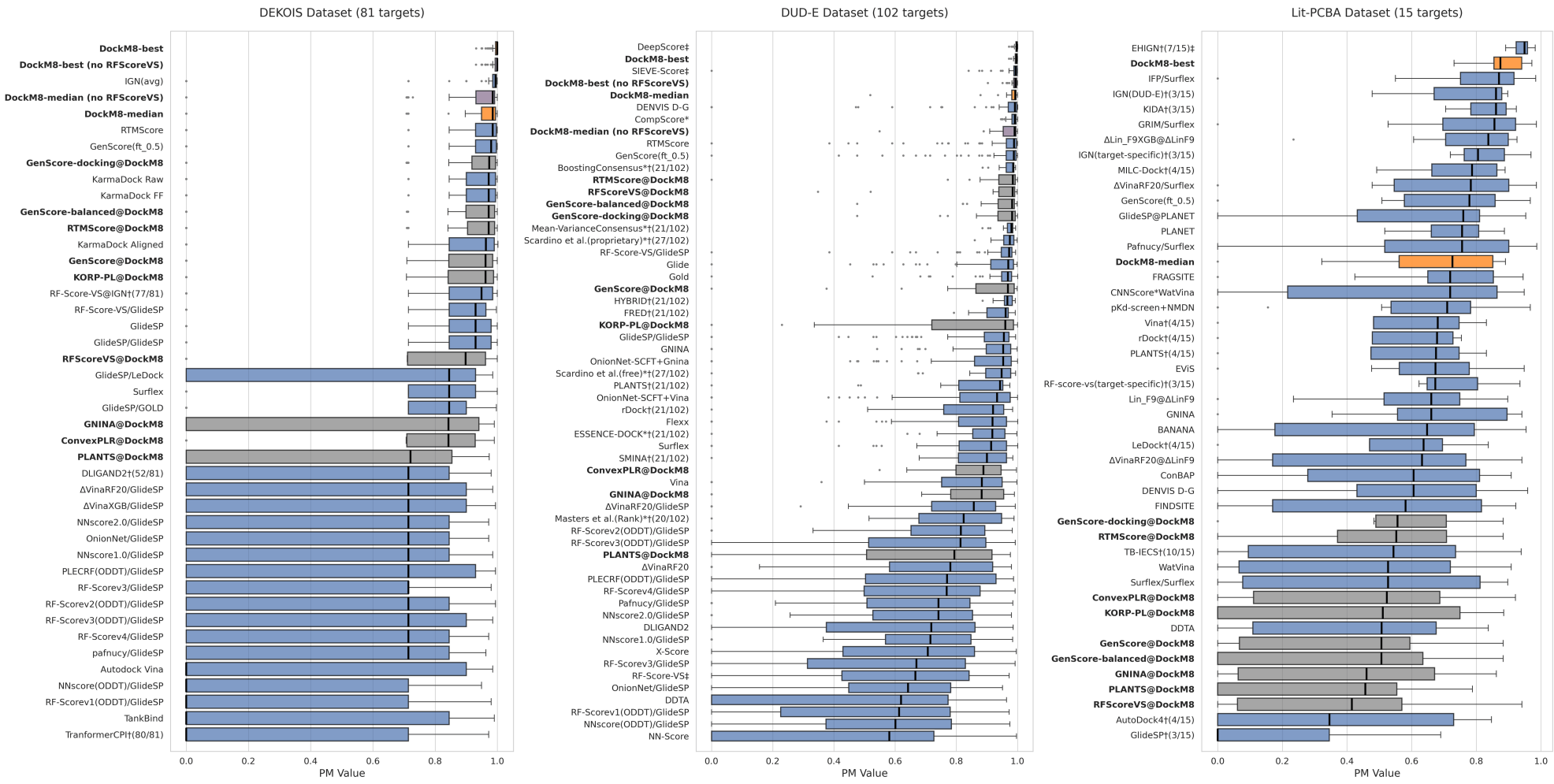

Fig. S9 Evaluation of PM at 1% including all published models and all targets in the datasets. See Section 1 for notation.

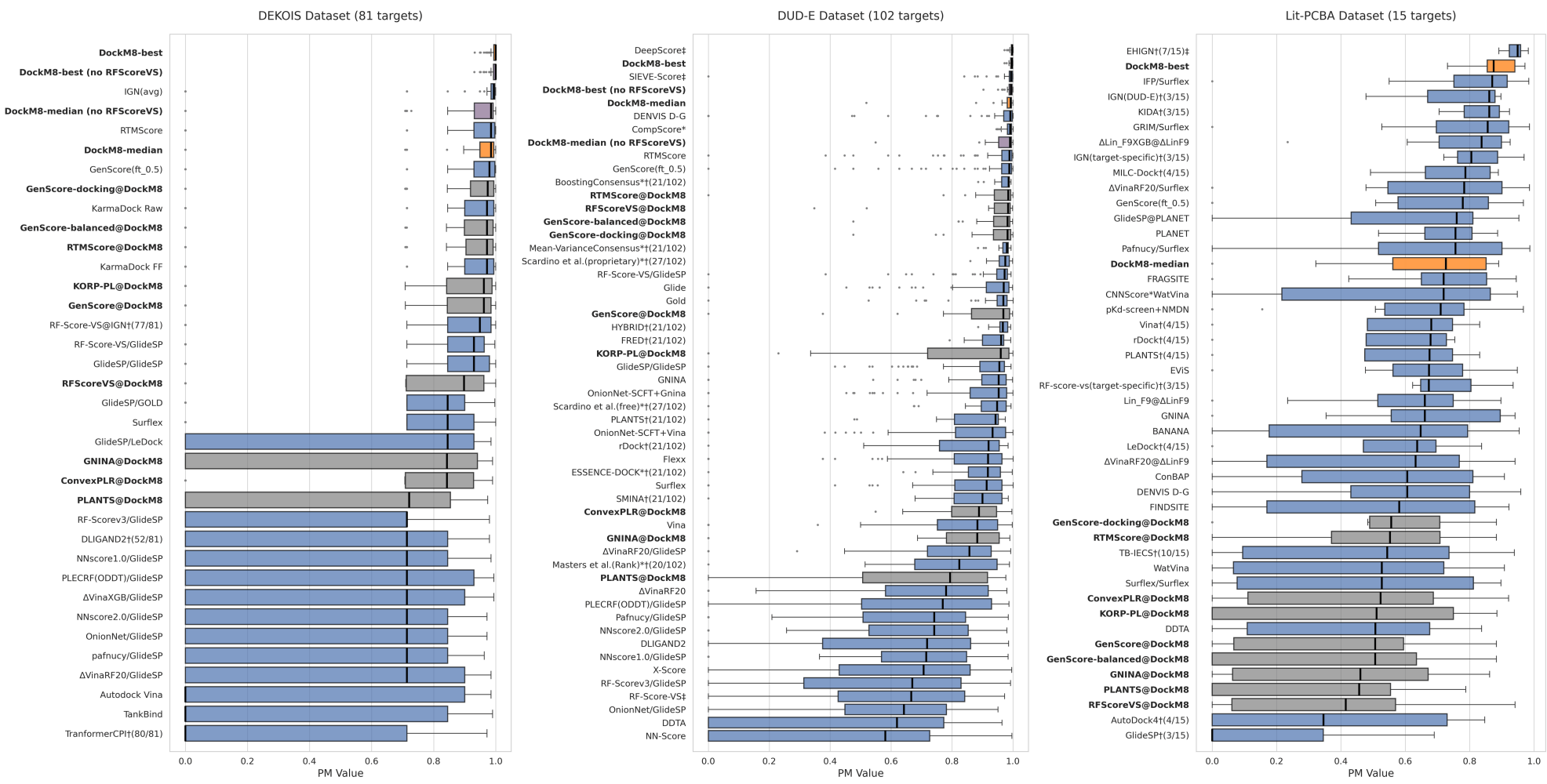

Fig. S10 Evaluation of PM at 1% including all targets in the datasets, not limited to those tested with DockM8. See Section 1 for notation.

1.5 Relative Enrichment Factor at 1%

REF at 1% results are shown across the four variants (Figures S11, S12, S13, and S14).

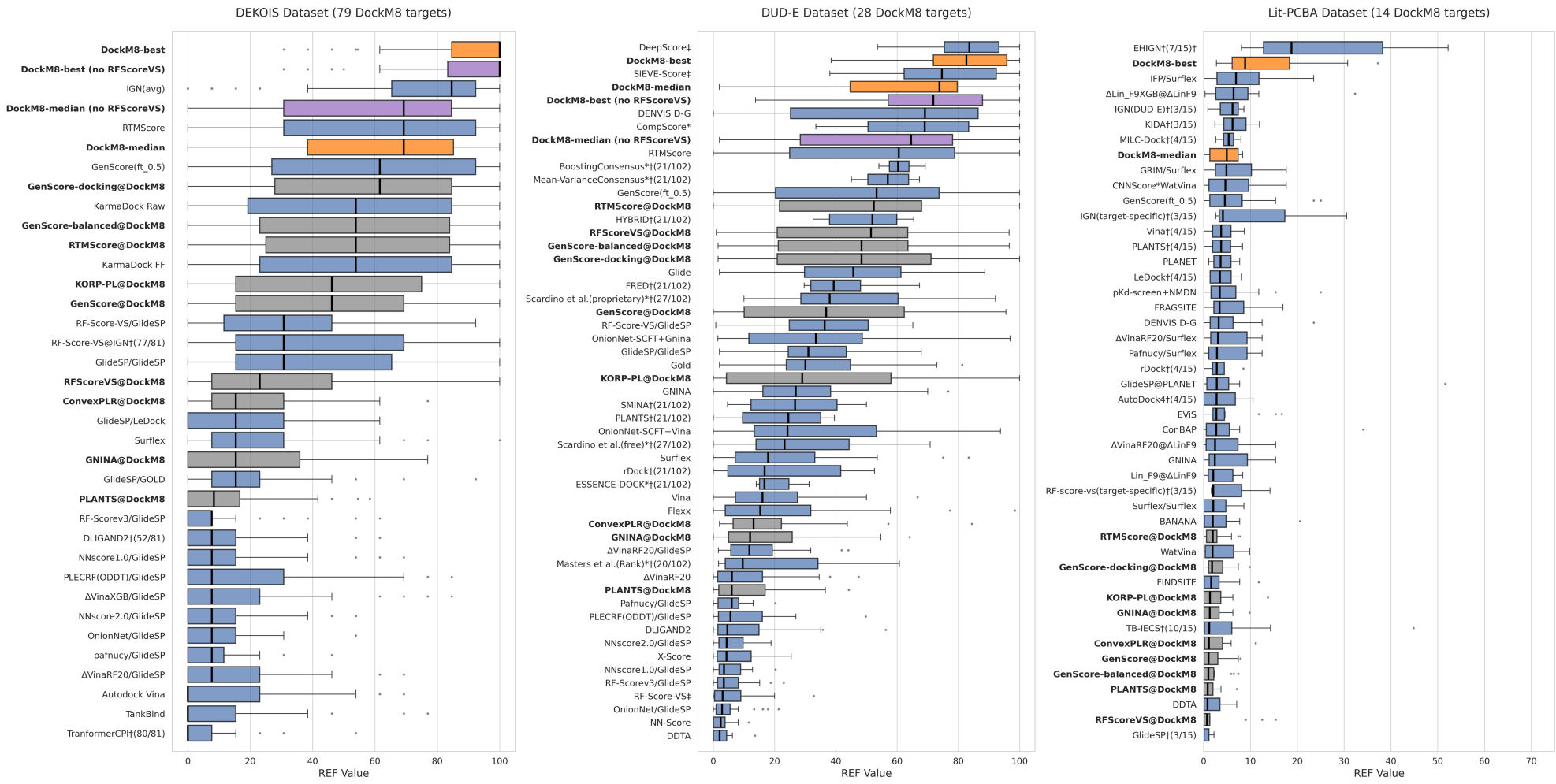

Fig. S11 Evaluation of REF at 1% of DockM8 compared to published protocols on the DEKOIS, DUD-E, and Lit-PCBA datasets using the standard target set. See Section 1 for notation.

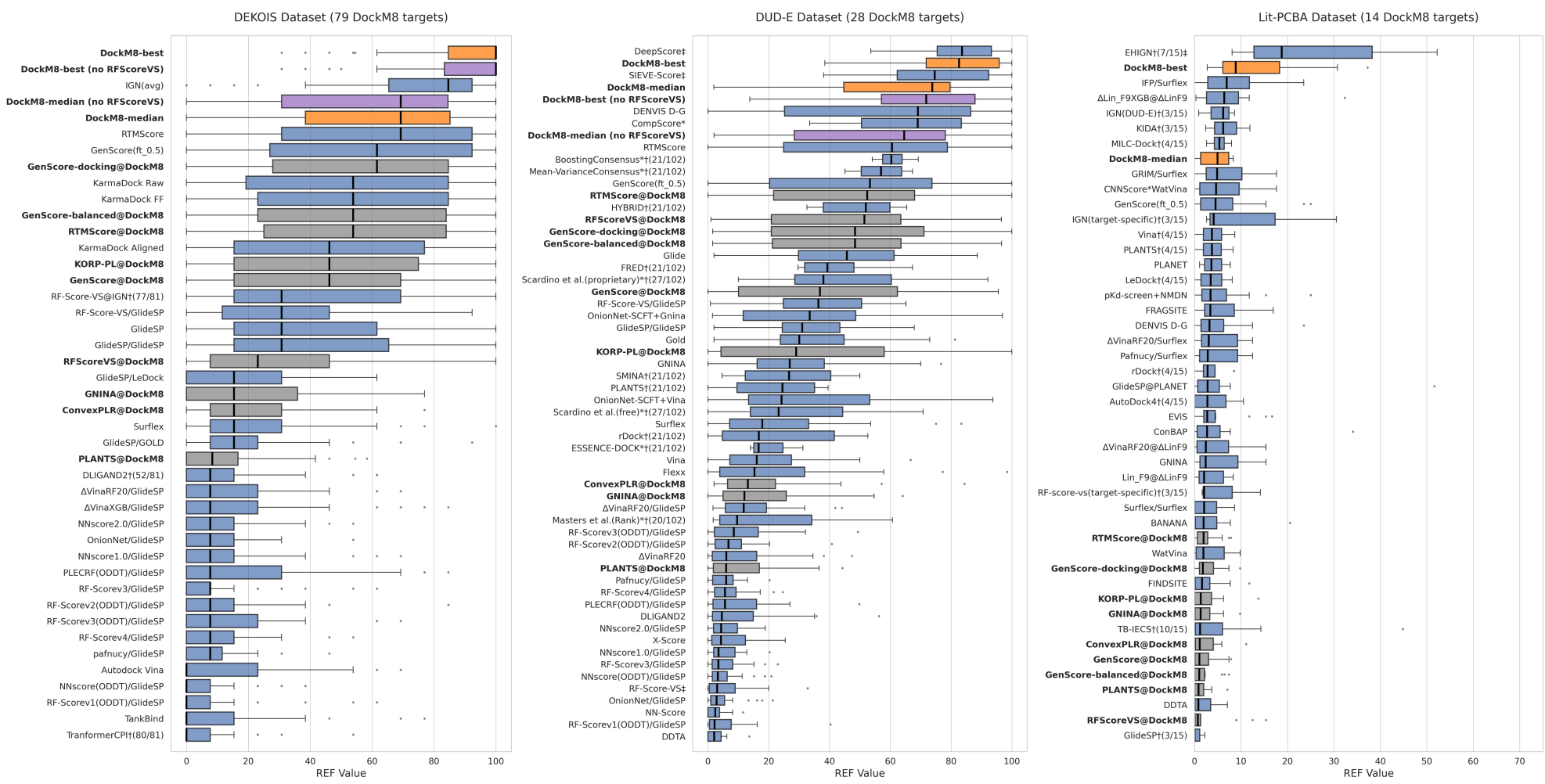

Fig. S12 Evaluation of REF at 1% including all published models for which literature data was obtained, using the standard target set. See Section 1 for notation.

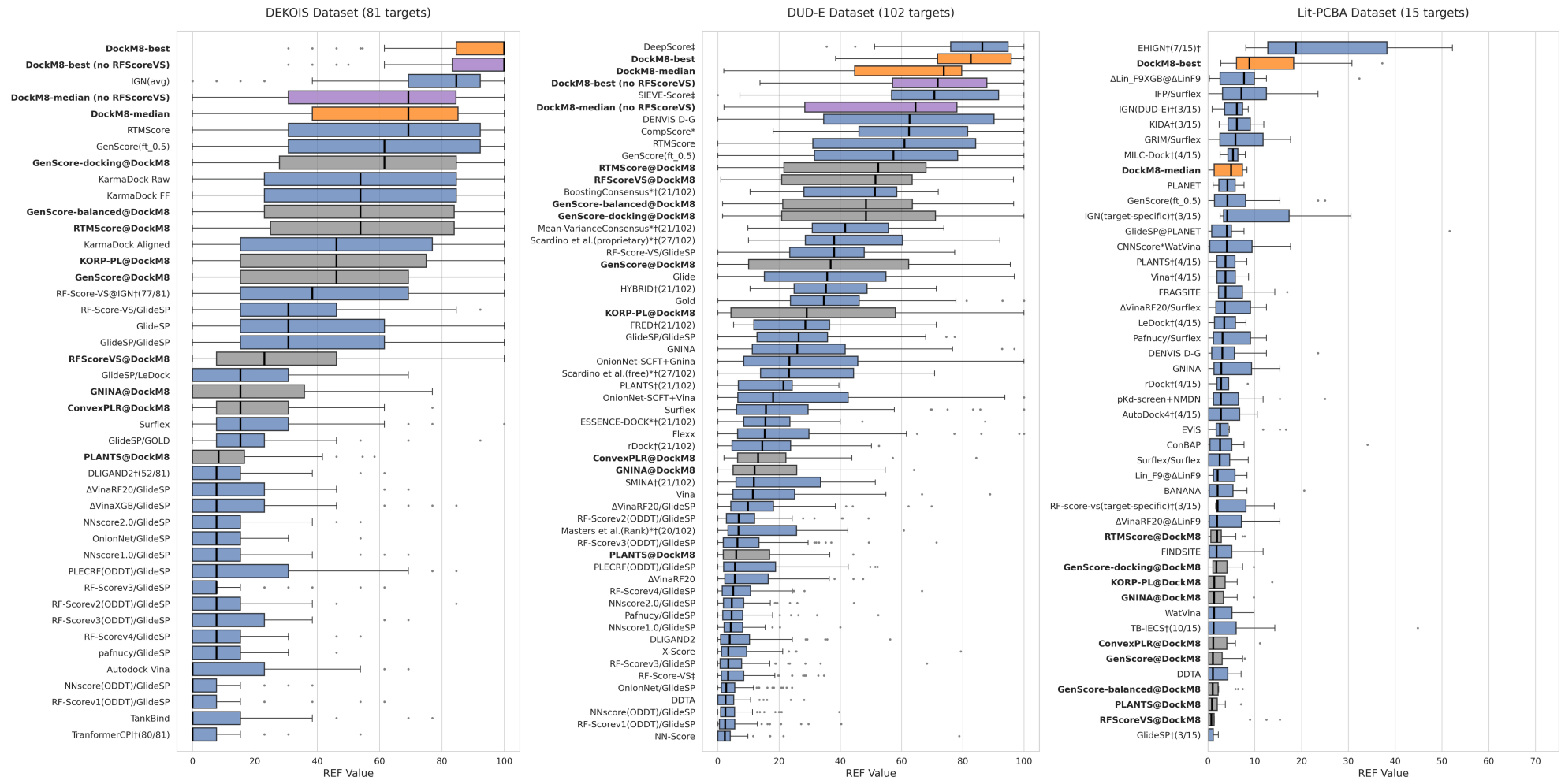

Fig. S13 Evaluation of REF at 1% including all published models and all targets in the datasets. See Section 1 for notation.

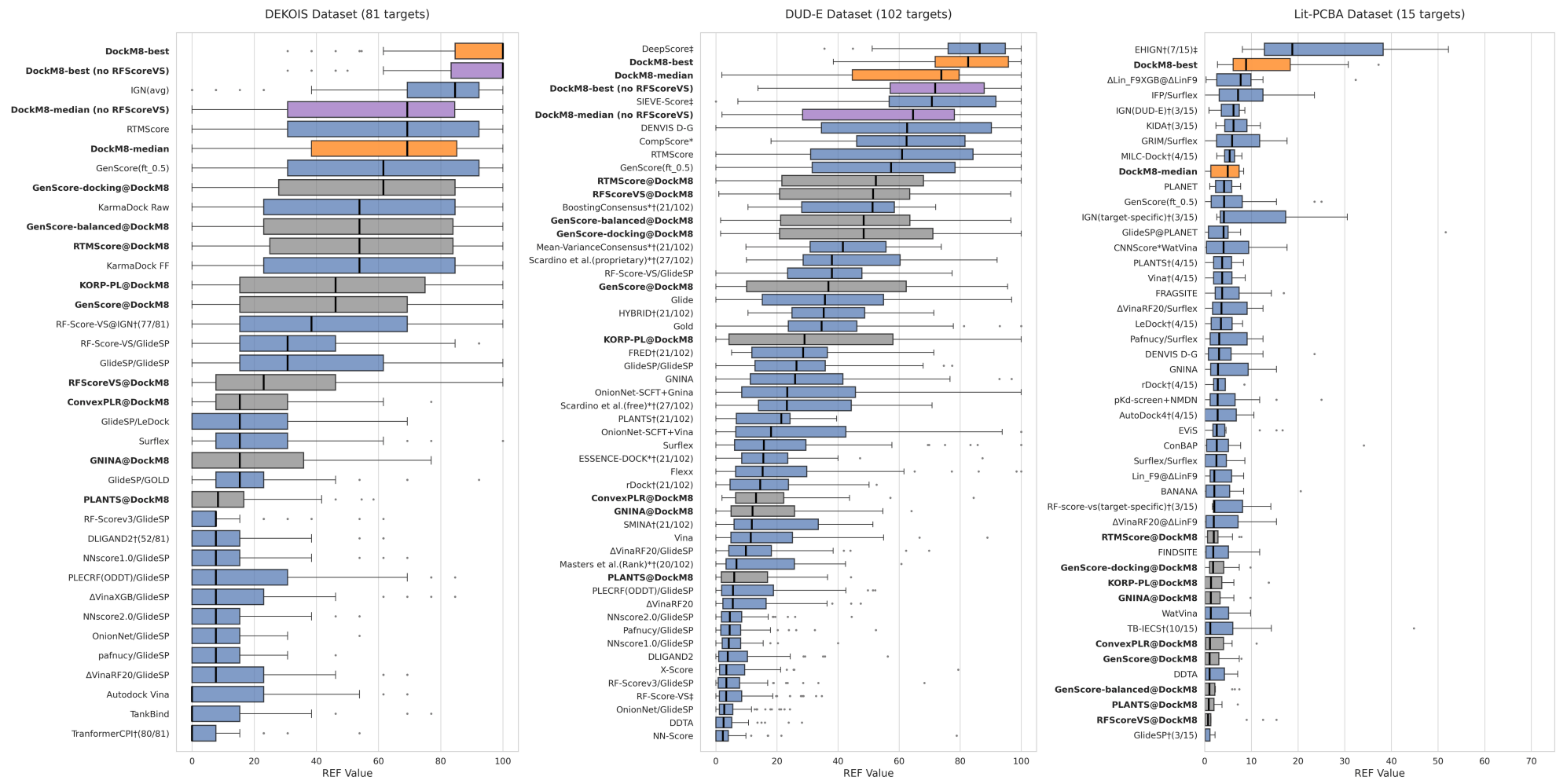

Fig. S14 Evaluation of REF at 1% including all targets in the datasets, not limited to those tested with DockM8. See Section 1 for notation.

2 Generalizability Assessment

Training vs. Validation AUC-ROC for all 14 Lit-PCBA targets under Asymmetric Validation Embedding (AVE) splits is shown in Figure S15.

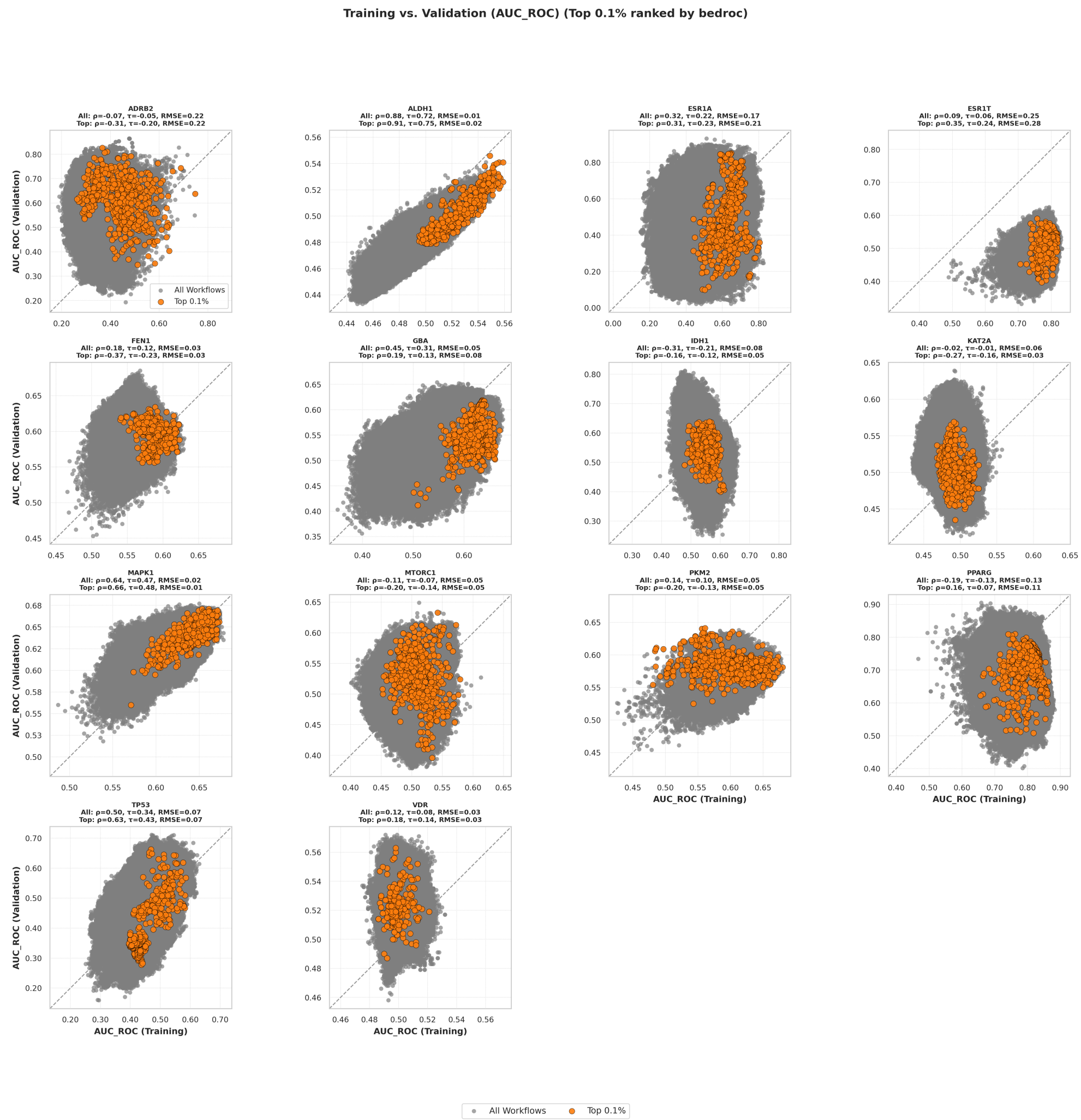

Fig. S15 Training vs. Validation AUC-ROC performance across all 14 Lit-PCBA targets using AVE splits. Each point represents a unique workflow configuration with gray circles showing all workflows and orange points highlighting the top 0.1% of workflows ranked by training-set BEDROC score. For each target, correlation metrics (Spearman's ρ, Kendall's τ, and RMSE) are displayed separately for all workflows (All) and the top 0.1% subset (Top).

The agreement between training and validation for the top-performing workflows relates to each target's active-to-inactive ratio, as shown in Figure S16.

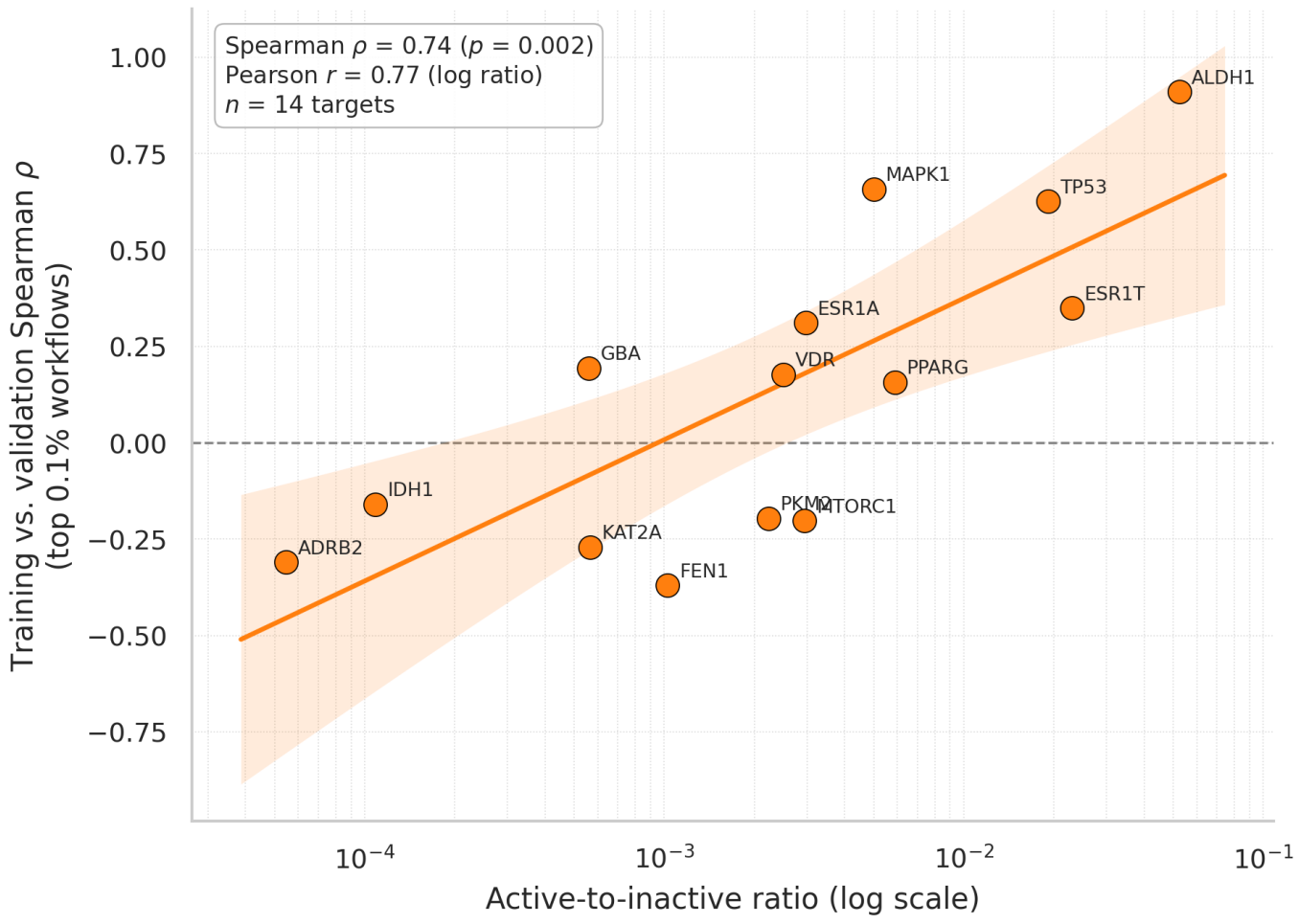

Fig. S16 Training vs. validation agreement (Spearman's ρ between training and validation AUC-ROC) for the top 0.1% of workflows per target, ranked by training-set BEDROC, as a function of each Lit-PCBA target's active-to-inactive ratio. Agreement increases with the ratio (Spearman $\rho = 0.74$, $p = 0.002$; $n = 14$ targets). The solid line is an ordinary-least-squares fit in log-ratio space with a 95% confidence band.
